# Gut microbial metabolite 4-hydroxybenzeneacetic acid drives colorectal cancer progression via accumulation of immunosuppressive PMN-MDSCs

**DOI:** 10.1172/JCI181243

**Published:** 2025-04-03

**Authors:** Qing Liao, Ximing Zhou, Ling Wu, Yuyi Yang, Xiaohui Zhu, Hangyu Liao, Yujie Zhang, Weidong Lian, Feifei Zhang, Hui Wang, Yanqing Ding, Liang Zhao

**Affiliations:** 1 Department of Pathology, Shunde Hospital of Southern Medical University, Foshan, China.; 2 Department of Pathology & Guangdong Province Key Laboratory of Molecular Tumor Pathology, Basic Medical College, Southern Medical University, Guangzhou, China.; 3 Department of Pathology, Nanfang Hospital, Southern Medical University, Guangzhou, China.; 4 Department of Plastic and Aesthetic Surgery, Nanfang Hospital, Southern Medical University, Guangzhou, China.; 5 Department of Medical Oncology, Affiliated Tumour Hospital of Guangzhou Medical University, Guangzhou, China.

**Keywords:** Gastroenterology, Immunology, Oncology, Cancer immunotherapy, Colorectal cancer

## Abstract

Colorectal cancer (CRC) is characterized by an immune-suppressive microenvironment that contributes to tumor progression and immunotherapy resistance. The gut microbiome produces diverse metabolites that feature unique mechanisms of interaction with host targets, yet the role of many metabolites in CRC remains poorly understood. In this study, the microbial metabolite 4-hydroxybenzeneacetic acid (4-HPA) promoted the infiltration of PMN myeloid-derived suppressor cells (PMN-MDSCs) in the tumor microenvironment, consequently inhibiting the antitumor response of CD8^+^ T cells and promoting CRC progression in vivo. Mechanistically, 4-HPA activates the JAK2/STAT3 pathway, which upregulates CXCL3 transcription, thereby recruiting PMN-MDSCs to the CRC microenvironment. Selective knockdown of CXCL3 resensitized tumors to anti-PD-1 immunotherapy in vivo. Chlorogenic acid reduces the production of 4-HPA by microbiota, likewise abolishing 4-HPA–mediated immunosuppression. The 4-HPA content in CRC tissues was notably increased in patients with advanced CRC. Overall, the gut microbiome uses 4-HPA as a messenger to control chemokine-dependent accumulation of PMN-MDSC cells and regulate antitumor immunity in CRC. Our findings provide a scientific basis for establishing clinical intervention strategies to reverse the tumor immune microenvironment and improve the efficacy of immunotherapy by reducing the interaction among intestinal microbiota, tumor cells, and tumor immune cells.

## Abstract 2

Gut microbiota disbiosis promoted colorectal tumorigenesis and shaped immunosuppressive microenvironment by producing 4-HPA. Targeting 4-HPA represent a novel strategy to reverse immunosuppressive microenvironment and enhance the response to immunotherapy in CRC patients.

## Graphical abstract

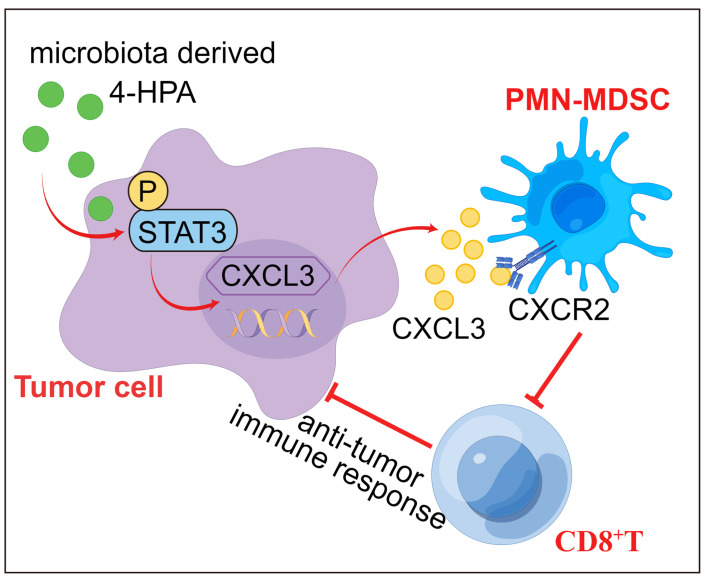

## Introduction

Colorectal cancer (CRC) is 1 of the most commonly diagnosed malignancies worldwide and is characterized by high morbidity and mortality rates (1). Although great progress has been made in the systemic treatment of CRC, variations in mortality rates among patients with CRC remain pronounced (2, 3). In recent years, immune checkpoint blockade (ICB) therapy has improved the clinical outcome of multiple malignancies (4, 5), including melanoma, br=east cancer, non–small cell lung cancer, and gastrointestinal malignancies (6–9). Despite these successes, ICB therapy is currently only applicable to patients with DNA mismatch repair-deficient/microsatellite instability-high metastatic CRC, representing approximately 15% of all CRC cases (10). Expanding the benefits of immunotherapy to a broader population of patients with CRC remains a major research challenge.

In recent years, the gut microbiota has emerged as a critical factor in regulating human health and disease (11). There is clear evidence for the role of gut microbiota in maintaining a well-balanced immune response by influencing the immune system both locally and systemically (12). Accumulating evidence suggests the gut microbiota influences the response of tumors to immunotherapy (13–15). Notably, gastrointestinal tumors are exposed to bacterial components and metabolites, and patients with CRC exhibit a dysbiotic gut microbiota (16). Microbial metabolites and virulence factors from the commensal microbiota have been highlighted as potentially playing a vital role in mediating the crosstalk between the microbiota and the host immune system (17–19). However, different metabolites may have unique functions, and their roles in CRC remain poorly understood.

Identifying the types of molecules produced by specific bacteria and elucidating the mechanisms by which they affect the tumor immune microenvironment present great challenges. In this study, changes in key metabolites, caused by dysbiosis of gut microbiota, were observed using nontargeted metabolomics. Interestingly, we found that a microbe-derived metabolite, 4-hydroxybenzeneacetic acid (4-HPA), is correlated with CXCL3 secretion in CRC. 4-HPA is associated with CRC, ulcerative colitis, Crohn’s disease, and lung cancer (20–22). Our study also revealed that 4-HPA levels were markedly increased in tumor tissues of patients with advanced CRC. To date, to our knowledge, the relationship between 4-HPA and the immune microenvironment in CRC has not been reported.

## Results

### Gut microbiota–induced cytokine secretion in CRC cells.

**Figure 1:**
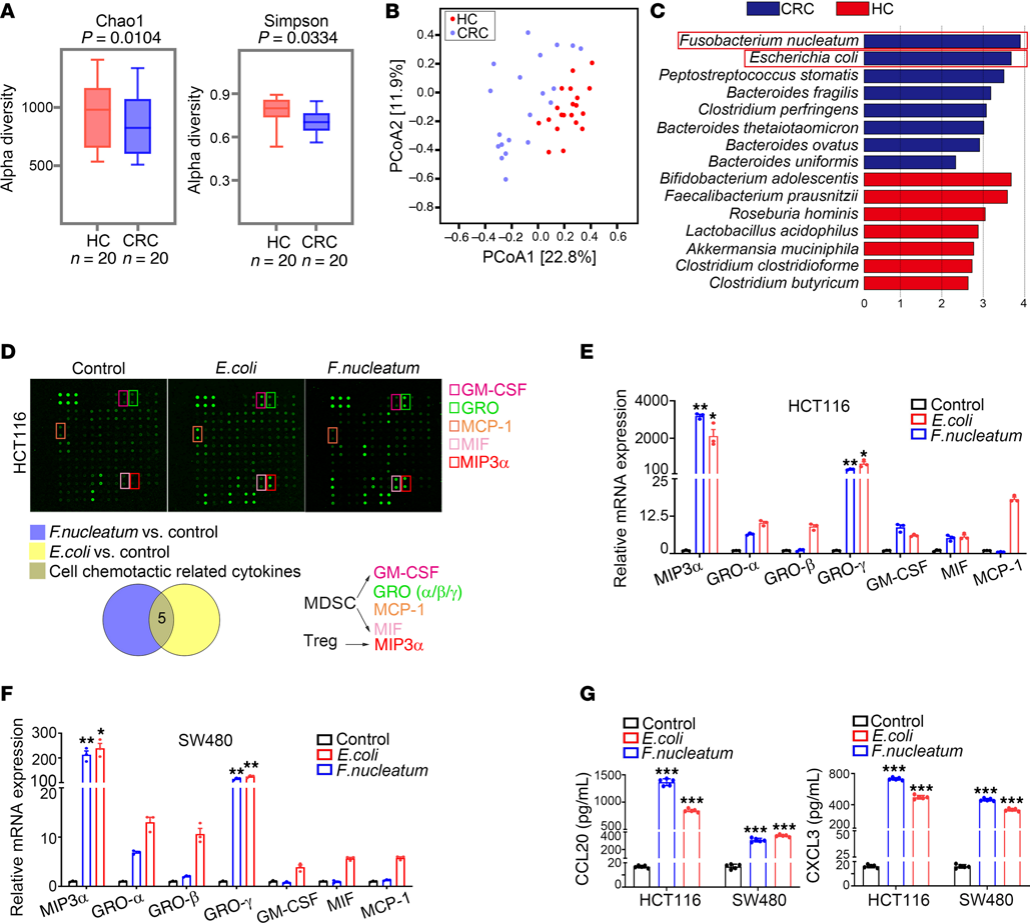
*F. nucleatum* and *E. coli* stimulated cytokine secretion in CRC cells. (**A**) α Diversity analysis using the Chao1 and the Simpson indexes in the CRC (*n* = 20) and HC (*n* = 20) groups. (**B**) Principal coordinate analysis at the species level between the CRC (*n* = 20) and HC (*n* = 20) groups. (**C**) The discriminant analysis effect size method identified marker species between the CRC (*n* = 20) and HC (*n* = 20) groups. Blue and red bars represent markers enriched in the CRC and HC groups, respectively. (**D**) A human cytokine antibody array was applied to detect the changes of inflammatory factors in CM of HCT116 cells treated with *F*. *nucleatum* or *E. coli*. Differential cytokines associated with immune cell chemotaxis are shown in the black boxes. A cytokine chip Wayne diagram is shown below those boxes. (**E** and **F**) qPCR analysis revealed changes in cytokine expression after co-culture with *F*. *nucleatum* and *E. coli* for 6 hours (*n* = 3). (**G**) ELISA detection of CCL20 and CXCL3 secretion from CRC cells after co-culture with *E. coli* and *F. nucleatum* (*n* = 5). All numerical data and error bars represent the mean ± SD of 3 independent experiments. Statistical analyses were conducted using 1-way ANOVA with Dunnett’s T3 correct multiple-comparison test. *P < 0.05, **P < 0.005, ***P < 0.0005.

To identify key gut microbes related to CRC, we performed fecal metagenomic sequencing of 20 patients with CRC and 20 healthy control participants (HCs) (Supplemental Table 2; supplemental material available online with this article; https://doi.org/10.1172/JCI181243DS1). Compared with the HCs, individuals with CRC exhibited markedly reduced α diversity, as shown by a decrease in the Chao1 and Simpson indexes (Figure 1A), indicating lower diversity of microbiota. β Diversity analysis (principal coordinate analysis) showed substantial differences in community structure between the 2 groups: the bacterial community structure of patients with CRC was separated from that of HC group, suggesting the composition of intestinal microbiota in the setting of CRC changed substantially (Figure 1B). Discriminant analysis effect size method revealed 15 differentially abundant species; among them, *Fusobacterium nucleatum* and *E. coli* were remarkably enriched in CRC (Figure 1C), consistent with previous analyses of the gut mucosal microbiome across stages of CRC study (23).

We then proceeded to evaluate the impact of these 2 bacteria on the CRC immune microenvironment. Cytokines play integral roles in tumor immunity, including heterotypic interactions between cancer cells and immune cells in the tumor microenvironment (TME) (24). To specifically identify the cytokines stimulated by *F. nucleatum* and *E. coli* in CRC cells, a human cytokine antibody chip was used to screen for secretion of 80 common cytokines and chemokines (25) (Supplemental Figure 1A and Supplemental Table 1). We found a series of cytokines whose concentrations were substantially higher than those in the control group, including chemotactic cytokines GM-CSF, GRO (GROα, GROβ, and GROγ), MCP-1, and MIF, which are related to the chemotaxis of myeloid-derived suppressor cells (MDSCs); CCL20, which is related to chemotaxis of CCR6^+^ Tregs; and angiogenin, which is related to angiogenesis (Figure 1D). In addition, quantitative PCR (qPCR) and ELISA verification showed that the cytokines with the most substantial changes were MIP-3α (CCL20) and GROγ (CXCL3) (Figure 1, E–G). Together, these results clearly show that *F. nucleatum* and *E. coli* markedly stimulated CRC cells to secrete CCL20 and CXCL3.

### Imbalance of gut microbiota mediates immunosuppressive microenvironment in CRC.

**Figure 2:**
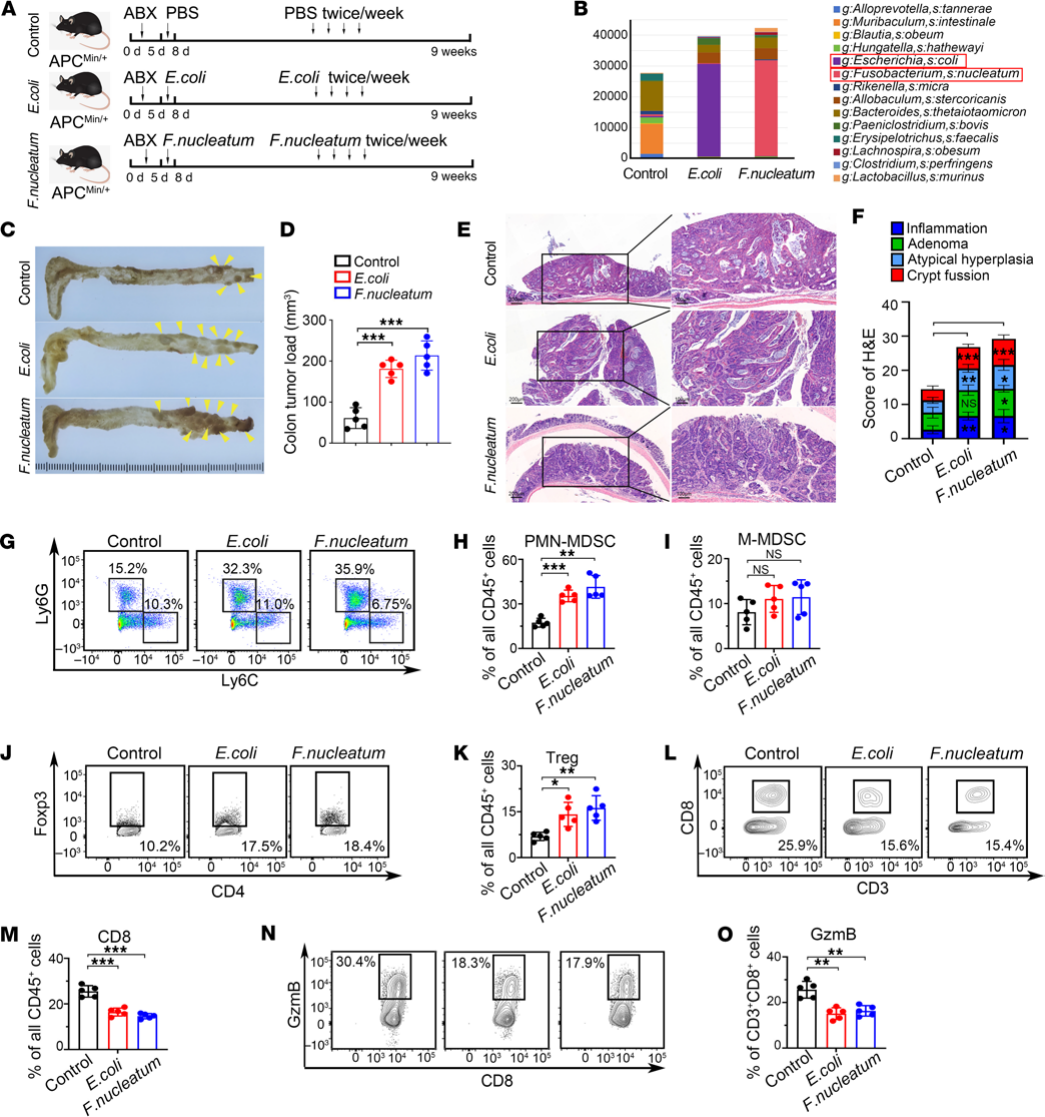
*F. nucleatum* and *E. coli* mediate immunosuppressive microenvironment in CRC. (**A**) Schematic diagram of the microbiota-treated Apc^min/+^ tumorigenesis mouse model administration method (*n* = 5). (**B**) Colonization efficiency of *E. coli* and *F. nucleatum* was assessed by metagenomic sequencing analysis of Apc^min/+^ mice after 8 days (5 days of antibiotics treatment to deplete their gut microbiota, followed by 3 days of *F. nucleatum*, *E*. *coli*, or PBS orally administration for microbiota colonization) . The relative abundance of Operational Taxonomic Units (OTUs) in fecal bacterial is shown. (**C**) Representative images of tumors in the intestines of Apc^min/+^ mice are shown. (**D**) Statistics of tumor load of the intestines derived from Apc^min/+^ mice treated with PBS, *E. coli*, or *F. nucleatum* (*n* = 5). (**E**) Representative images of tumorigenesis of intestines in Apc^min/+^ mice visualized by H&E staining. (**F**) H&E scoring of tumor-related lesions (including inflammation, adenoma, atypical hyperplasia, and crypt fusion) (n =5). (**G**) The percentages of PMN-MDSCs (CD11b^+^Ly6G^+^Ly6C^low^) and M-MDSCs (CD11b^+^Ly6G^-^Ly6C^hi^) in TILs (CD45^+^) of Apc^min/+^ mice were determined by flow cytometry sorting. (**H** and **I**) Statistical chart of PMN-MDSCs and M-MDSCs (*n* = 5). (**J**) The percentage of Tregs (CD4^+^Foxp3^+^) in TILs (CD45^+^) of Apc^min/+^ mice, detected by flow cytometry sorting. (**K**) Statistical chart of Tregs (*n* = 5). (**L**) Tumor-infiltrating CD8^+^T cells in TILs (CD45^+^) of Apc^min/+^ mice, detected by flow cytometry sorting. (M). Statistical chart of CD8^+^T cells (*n* = 5). (**N**) The granule productions (GzmB^+^) of CD8^+^T cells. (**O**) Statistical chart of GzmB^+^ cells (*n* = 5). Data and error bars represent the mean ± SD of 3 independent experiments. Statistical analyses were conducted using 1-way ANOVA with Dunnett’s T3 correct multiple-comparison test. *P < 0.05, **P < 0.005, ***P < 0.0005.

To elucidate the role of intestinal microbiome imbalance of *E. coli* and *F. nucleatum* in the biological behavior of CRC, Apc^min/+^ tumorigenesis mouse models and orthotopic implanted CRC mouse models were constructed (26). An antibiotic cocktail consisting of vancomycin, neomycin, streptomycin, and metronidazole was added to the drinking water of the mice to deplete gut commensal bacteria (27). The mice were then administered *E. coli*, *F. nucleatum*, or PBS (Figure 2A and Supplemental Figure 2A). Successful microbiota colonization with *E. coli* or *F. nucleatum* was confirmed (Figure 2B and Supplemental Figure 2B).

The imbalance of *E. coli* or *F. nucleatum* increased the tumor load (Figure 2, C and D) and exacerbated the severity of tumor-related lesions (Figure 2, E and F) in Apc^min/+^ mice. In the orthotopic implanted CRC mouse model, *E. coli–*treated mice and *F. nucleatum–*treated mice had a reduced survival rate (Supplemental Figure 2C), enhanced tumorigenic capacity of CRC cells (Supplemental Figure 2, D–F), and liver metastasis was promoted (Supplemental Figure 2, G–I), compared with controls. Tumor-infiltrating lymphocytes (TILs) in mice were detected using flow cytometry (Supplemental Figure 3, A–C). In both the Apc^min/+^ tumorigenesis mouse model and orthotopic implanted CRC mouse model, the imbalance of *E. coli* or *F. nucleatum* promoted the infiltration of PMN myeloid-derived suppressor cells (PMN-MDSCs) and Tregs in the tumor (Figure 2, G, H, J, and K and Supplemental Figure 3, D and F) and decreased the infiltrating and antitumor function of CD8^+^ T cells (Figure 2, L–O and Supplemental Figure 3, G and H). However, there was no statistically significant difference in the effect on monocytic MDSCs (M-MDSCs) (Figure 2I and Supplemental Figure 3E).

PMN-MDSCs and Tregs are important negative modulators of tumor immunity. IHC showed that the imbalance of *E. coli* or *F. nucleatum* promoted the infiltration of MDSCs and Tregs in the tumor and decreased the infiltration of CD8^+^ T cells (Supplemental Figure 3, I and J). Collectively, our results indicate the imbalance of *E. coli* or *F. nucleatum* caused the accumulation of immunosuppressive cells (PMN-MDSCs and Tregs) and inhibited the antitumor response of CD8^+^ T cells, thereby fostering an immunosuppressive microenvironment.

### CXCL3 is the key cytokine of tumor immunosuppression caused by microbiota dysbiosis in CRC.

**Figure 3:**
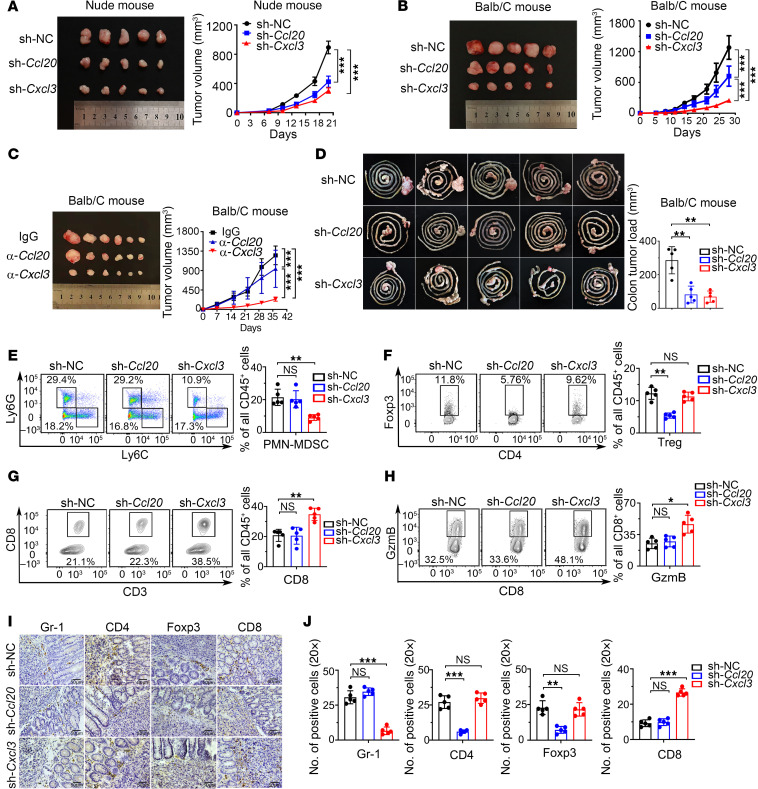
Knockdown CXCL3 inhibits tumor growth by preventing PMN-MDSC accumulation and activating CD8^+^ T-cell infiltration. (**A**) Knockdown of *Ccl20* and *Cxcl3* inhibits the growth of CT26 subcutaneous tumors in nude mice (*n* = 5). A photograph of CT26 subcutaneous tumors in nude mice and a graph of tumor growth are shown. (**B**) Knockdown of *Ccl20* and *Cxcl3* inhibits the growth of CT26 subcutaneous tumors in BALB/c mice (*n* = 5). A photograph of CT26 subcutaneous tumors in BALB/c mice and a graph of tumor growth are shown. (**C**) Neutralizing antibodies of *Ccl20* and *Cxcl3* inhibited subcutaneous tumors in BALB/c mice(*n* = 6). A photograph of CT26 subcutaneous tumors in BALB/c mice and a graph of tumor growth are shown. (**D**) Knockdown of *Ccl20* and *Cxcl3* inhibits the progression of CT26 orthotopic implanted tumor in BALB/c mice (*n* = 5). Representative tumor images and tumor load are shown. (**E**) The percentage of PMN-MDSCs (CD11b^+^Ly6G^+^Ly6C^low^) in TILs (CD45^+^) of orthotopic implanted CRC mice detected by flow cytometry sorting. A bar chart indicating statistical values is presented (*n* = 5). (**F**) The percentage of Tregs (CD4^+^Foxp3^+^) in TILs (CD45^+^) of orthotopic implanted CRC mice detected by flow cytometry sorting. A bar chart indicating statistical values is presented (*n* = 5). (**G** and **H**) Tumor-infiltrating CD8^+^T cells and their granule production (GzmB^+^) in TILs (CD45^+^) of orthotopic implanted CRC mice detected by flow cytometry sorting. Bar charts indicating statistical values are presented (*n* = 5). (**I**) MDSCs (Gr-1^+^), Tregs (CD4^+^, Foxp3^+^), and CD8^+^ T-cell infiltration in tumor tissues of orthotopic implanted CRC mice. Representative IHC images are shown. (**J**) Histogram showing the number of Gr-1^+^, CD4^+^, Foxp3^+^, and CD8^+^ cells per ×20 objective lens visual field (*n* = 5). Data represent the mean ± SD of 3 independent experiments. We used 2-way ANOVA to determine statistical significance of subcutaneous tumor volume. The remaining statistical methods were conducted using 1-way ANOVA with Dunnett’s T3 correct multiple-comparison test. *P < 0.05, **P < 0.005, ***P < 0.0005. sh, short hairpin.

To explore the potential biological functions of CCL20 and CXCL3 in CRC, we constructed stable knockdown of *Ccl20* and *Cxcl3* in mouse CRC cell line CT26 (Supplemental Figure 4, A and B). Subcutaneous tumor models were established in T cell–deficient BALB/c nude mice and BALB/c mice. Knockdown of *Ccl20* and *Cxcl3* notably decreased tumor growth in nude mice, but there was no substantial difference between the short hairpin *Cxcl3* (sh-*Cxcl3*) and sh-*Ccl20* groups (Figure 3A). Notably, in BALB/c mice, the growth of subcutaneous tumors was reduced by knocking down *Cxcl3* and *Ccl20*, with *Cxcl3* having a stronger inhibitory effect than *Ccl20* (Figure 3B). Neutralizing antibodies against *Cxcl3* and *Ccl20* were used to explore the role of the cytokines secreted into the microenvironment. Similarly, anti-*Cxcl3* showed stronger antitumor activity than anti-*Ccl20* in BALB/c mice with normal immune function (Figure 3C).

These results prompted us to investigate the immune response in tumor burden. We hypothesized that interference with *Cxcl3* and *Ccl20* may cause differences in T-cell immune function, which may influence tumor formation. Therefore, we focused on the role of CXCL3 in the development of CRC. To explore the immune response of CCL20 and CXCL3, we constructed stable knockdown of *Ccl20* and *Cxcl3* in orthotopic implanted tumor mouse models (Figure 3D). Infiltrating lymphocyte in situ tumors were isolated and detected by flow cytometry. Knockdown of *Cxcl3* markedly suppressed the infiltration of PMN-MDSCs (Figure 3E), whereas there was no substantial difference in M-MDSCs (Supplemental Figure 4C). Knockdown of *Ccl20* inhibited the infiltration of Tregs (Figure 3F). Interestingly, knockdown of *Cxcl3*, but not of *Ccl20*, notably increased the frequency and activity of tumor-infiltrating granzyme B^+^ (GzmB^+^) CD8^+^ T cells (Figure 3, G and H). Tumor-infiltrating MDSCs, Tregs, and CD8^+^ T cells were further verified using IHC. Similarly, IHC revealed that sh-*Cxcl3*/MDSCs, but not *Ccl20*/Tregs, markedly increased the frequency of CD8^+^ T cells in the tumors (Figure 3, I and J). Collectively, these results clearly demonstrate that sh-*Cxcl3* inhibits PMN-MDSC infiltration and promotes CD8^+^ T-cell accumulation and antitumor function in CRC, thus inhibiting the progression of CRC. These data support the view that CXCL3 is the key cytokine mediating immunosuppression in CRC.

### The CXCL3/CXCR2 axis mediates MDSCs recruitment and inhibits T-cell effector function.

**Figure 4:**
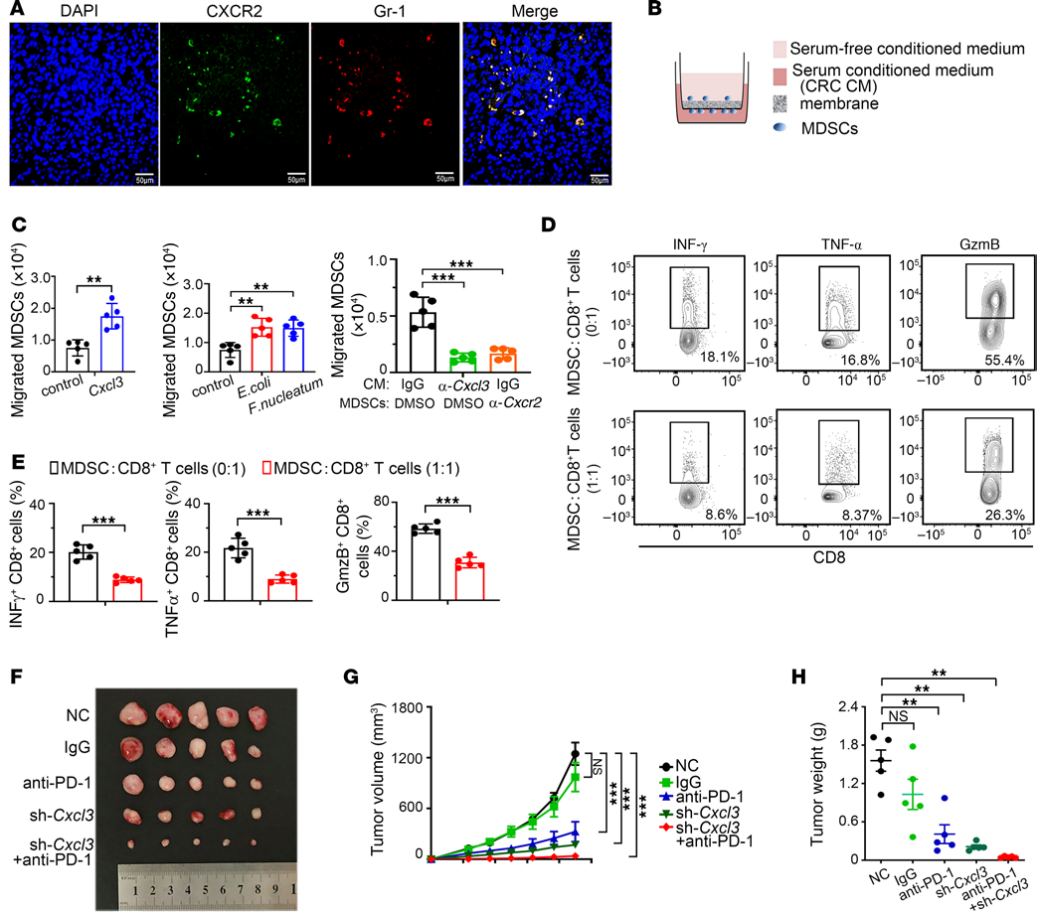
The CXCL3/CXCR2 axis mediates MDSC recruitment and inhibits T-cell effector function. (**A**) IF assays were performed to detect CXCR2 and Gr-1 in orthotopic cecal tumor of BALB/c mice. Scale bar: 50 μm. (**B** and **C**) The CXCL3-CXCR2 axis promoted the migratory abilities of MDSCs, as detected by transwell assays (*n* = 5). (**D** and **E**) Representative flow cytometry data show that MDSCs cells isolated from C57 mice inhibited cytokine and cytolytic granule production in CD8^+^T cells (**D**); the summarized result is presented in (**E**) (*n* = 5). (**F**) Effect of short hairpin *Cxcl3* (sh-*Cxcl3*) and PD-1 immunotherapy on subcutaneous tumor of BALB/c mouse: CT26 subcutaneous tumors (*n* = 5). The CD279 anti–PD-1 antibody or isotype control (IgG) was i.p. injected three times daily (**G** and **H**) Tumor growth (**G**) and weight (**H**) were monitored (*n* = 5). Data represent the mean ± SD of 3 independent experiments. Statistical analyses were conducted using Student’s *t* test (2-comparison test) and 1-way ANOVA with Dunnett’s T3 correct multiple-comparison test. We used 2-way ANOVA to determine statistical significance of tumor volume. *P < 0.05; **P < 0.005; ***P < 0.0005.

PMN-MDSCs are primarily recruited by members of the angiogenic CXC chemokine family members. The homologous receptor of CXCL3 is CXCR2, which is crucial for the migration of PMN-MDSCs from the bone marrow to the tumor (28). We hypothesized that the CXCL3/CXCR2 axis plays a key role in shaping the immune microenvironment of CRC. Immunofluorescence (IF) assays were used to detect the expression and co-localization of cxcr2 and Gr-1 in CRC. The results showed that MDSCs infiltrating in mouse CRC tissues predominantly expressed CXCR2 (Figure 4A). The chemotactic effect of the CXCL3/CXCR2 axis on MDSCs was evaluated using a transwell assay in vitro. *F. nucleatum*, *E. coli,* and overexpressed CXCL3 substantially upregulated the expression of CXCL3 (Supplemental Figure 5A). *F. nucleatum*, *E. coli*, and overexpressed *Cxcl3–*treated CT26 cells substantially promoted MDSC migration, whereas neutralizing antibodies against CXCL3 and CXCR2 markedly inhibited MDSC migration (Figure 4, B and C, and Supplemental Figure 5B). We performed co-culture experiments to verify the correlation between MDSCs and CD8^+^ T-cell function. The results showed that GzmB, IFN-γ, and TNF-α levels were notably decreased in the MDSCs co-culture group, indicating that MDSCs strongly inhibited the cytotoxic function of CD8^+^ T cells (Figure 4, D and E, and Supplemental Figure 5, C and D).

To clarify the effect of CXCL3 on ICB therapy, we established a subcutaneous tumor model in BALB/c mice. The growth of subcutaneous tumors in mice treated with anti–PD-1 antibody combined with *Cxcl3* knockdown was substantially lower than that in mice treated with sh-*Cxcl3* or anti–PD-1 antibody alone (Figure 4, F–H). Together, these results suggest the CXCL3/CXCR2/MDSCs axis inhibits CD8^+^ T-cell functions and that regulating the CXCL3-CXCR2 axis enhances the efficacy of PD-1 therapy.

### The microbial metabolite 4-HPA stimulates CRC cells to secrete CXCL3.

**Figure 5:**
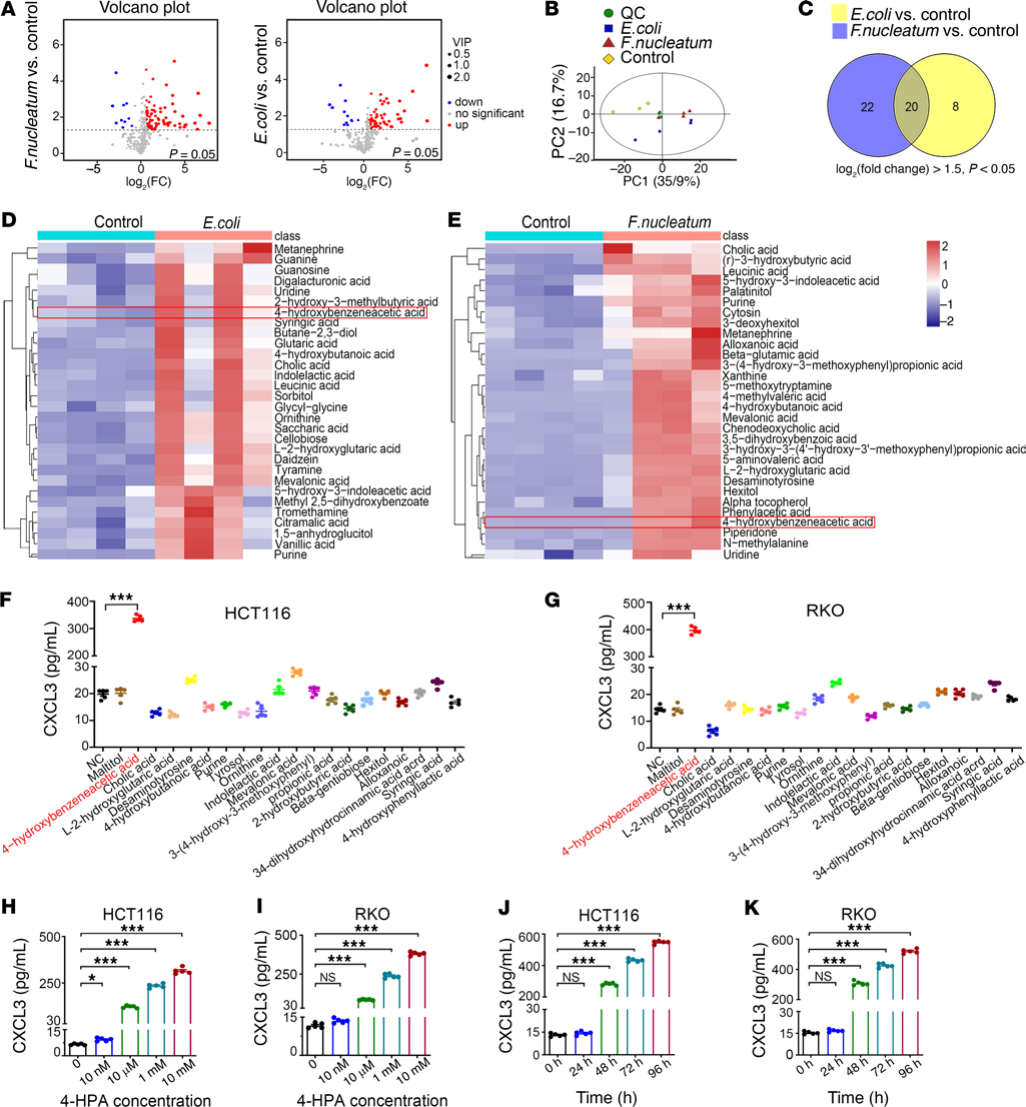
Nontargeted metabolomics reveal key metabolites that mediate CXCL3 secretion. (**A**) Identification of differential metabolites in the *E*. *coli* and *F*. *nucleatum* imbalance models using nontargeted metabolomics, presented volcano plots. (**B**) Principal component analysis (PCA) comparing the *E*. *coli* or *F*. *nucleatum* imbalance groups with the control group. (**C**) A Wayne chart illustrating differential metabolites between the *E*. *coli* or *F*. *nucleatum* imbalance models and the control group. (**D** and **E**) Heatmaps depicting differential metabolites in the *E*. *coli* (**D**) and *F*. *nucleatum* (**E**) imbalance groups compared with the control groups. *P* < 0.05, 2-tailed Mann-Whitney *U* test. (**F** and **G**) ELISA was used to assess the impact of differential metabolites (1 mM; 48 hours) on CXCL3 levels (*n* = 5). (**H**–**K**) ELISA assays measuring the effects of the 4-HPA concentration gradient and time gradient on CXCL3 secretion (*n* = 5). Data represent the mean ± SD of 3 independent experiments. Statistical analyses were conducted using 1-way ANOVA with Dunnett’s T3 correct multiple-comparison test. *P < 0.05, ***P < 0.0005. PC2, principal components 2; QC, quality control; VIP, variable importance in projection.

Accumulating evidence suggests that some commensal microbiota modulates the host immune system via microbial metabolites. However, the underlying molecular mechanisms remain unclear. To further identify differential metabolites caused by the imbalance of *E. coli* and *F. nucleatum*, we used nontargeted gas chromatography–tandem mass spectrometry (GC-MS/MS) to analyze metabolites derived from *E*. *coli* or *F*. *nucleatum* imbalanced mouse models (Figure 5, A and B). We found that 20 metabolites were notably elevated in *E. coli* and *F. nucleatum* imbalance models (log_2_ fold change > 1.5 and *P* < 0.05) (Figure 5, C–E). To determine whether these metabolites influenced cytokine secretion, we stimulated HCT116 and RKO cells with various metabolites and measured CXCL3 levels using ELISA. We found that 4-HPA substantially stimulated CXCL3 secretion in CRC cells (Figure 5, F and G). Therefore, we focused on the microbiota-dependent metabolite 4-HPA. Additional ELISA analyses revealed that 4-HPA stimulated CXCL3 secretion in a concentration- and time-dependent manner (Figure 5, H–K). In conclusion, our nontargeted metabolomics approach using GC-MS/MS revealed that the bacterial metabolite 4-HPA promotes the secretion of CXCL3 in CRC.

### 4-HPA promotes the transcriptional regulation of CXCL3 by STAT3 in CRC cells.

**Figure 6:**
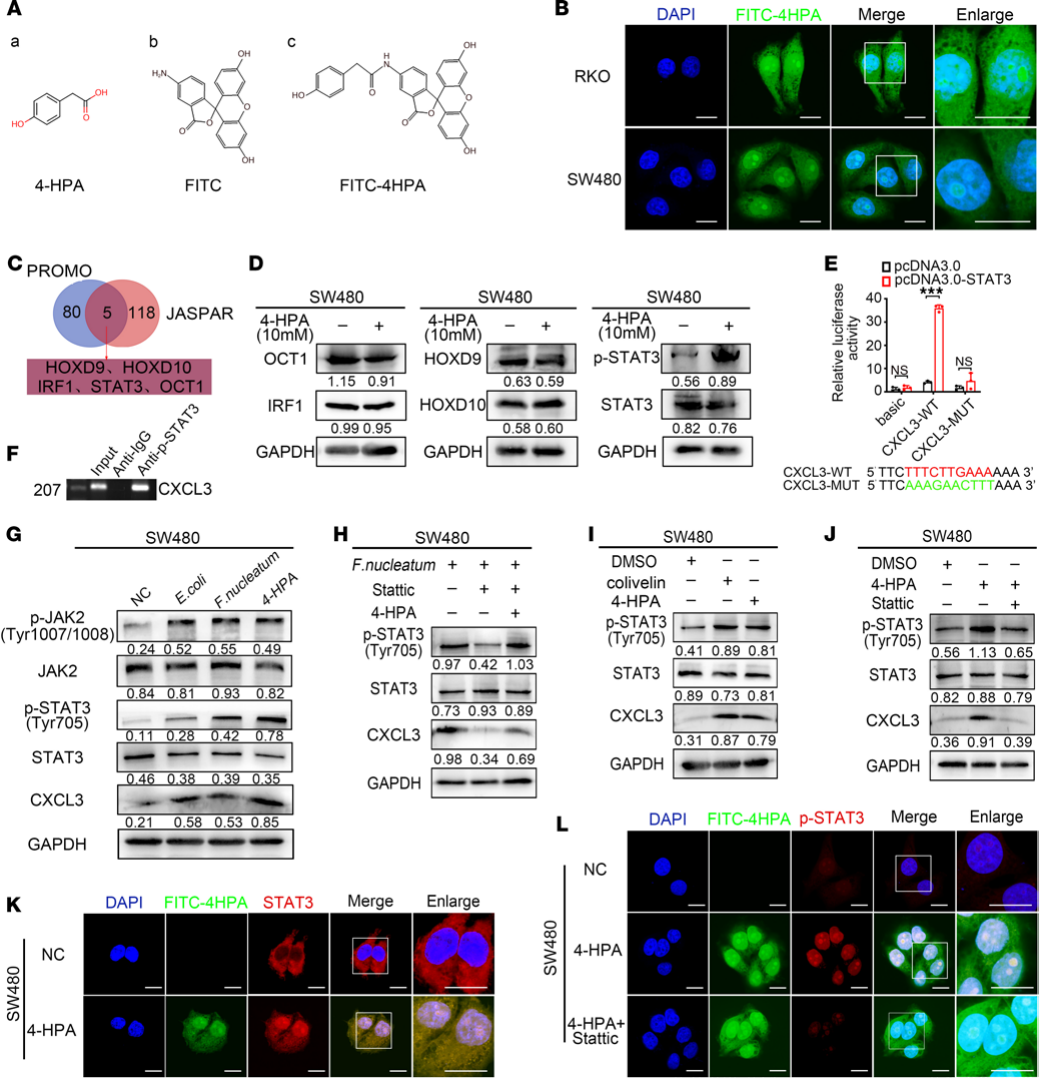
4-HPA promotes the transcription of CXCL3 regulated by STAT3 in CRC cells. (**A**) Chemical structures of 4-HPA, FITC, and FITC-labeled -HPA. (**B**) Detection of FITC-labeled 4-HPA by fluorescence confocal microscopy in RKO and SW480 cells. Scale bar: 50 μm. (**C**) Predicted transcription factors for CXCL3. (**D**) WB analysis of the effects of 4-HPA on transcription factors in CRC cell lines. (**E**) The binding sites of STAT3 and CXCL3 were confirmed using a dual-luciferase reporter assay (*n* = 3). (**F**) Transcriptional regulation of CXCL3 by p-STAT3 was detected using ChIP assays. (**G**) WB analysis of the JAK2/STAT3 signaling pathway and CXCL3 in SW480 cells. (**H**–**J**) WB analysis of CXCL3 in SW480 cells. (**K** and **L**) IF assays visualizing the subcellular localization of STAT3 and p-STAT3 in SW480 cells treated with 4-HPA. Scale bar: 50 μm. Data represent the mean ± SD of 3 independent experiments. Statistical analyses were conducted using Student’s *t* test. ***P < 0.0005. MUT, mutation; NC, negative control.

To explore the molecular mechanism by which 4-HPA promotes the secretion of CXCL3, we constructed FITC-labeled 4-HPA to detect its localization in CRC cells (Figure 6A). FITC-labeled 4-HPA was detected in both the cytoplasm and nucleus of CRC cells (Figure 6B). To further explore the mechanism by which 4-HPA regulates CXCL3, we used the PROMO tool and JASPAR database to predict transcription factors that could bind to the CXCL3 promoter. Considering the predicted intersection of the 2 sites, we preliminarily selected 5 transcription factors that can regulate CXCL3: OCT1, IRF1, HOXD9, HOXD10, and STAT3 (Figure 6C). Western blot (WB) analysis verified that 4-HPA enhanced the phosphorylation of STAT3 but did not affect other transcription factors (Figure 6D and Supplemental Figure 6A). ChIP assays demonstrated that STAT3 could bind to the CXCL3 promoters, whose binding sites were confirmed using a dual-luciferase reporter system (Figure 6, E and F).

We sought to determine the mechanism by which 4-HPA regulates CXCL3 and hypothesized that 4-HPA promotes STAT3 phosphorylation by activating the JAK2/STAT3 pathway. WB assays indicated that *E. coli*, *F. nucleatum*, and 4-HPA activated the JAK2/STAT3 pathway and upregulated CXCL3 (Figure 6G and Supplemental Figure 6C). An additional WB assay revealed that the STAT3 inhibiter Stattic inhibited the activation of STAT3 and the expression of CXCL3 stimulated by bacteria, whereas the introduction of 4-HPA notably abolished the inhibitory effects of Stattic (Figure 6H and Supplemental Figure 6D). Gain-of-function assays showed that STAT3 activator colivelin and 4-HPA promoted the phosphorylation of STAT3 and upregulated CXCL3 (Figure 6I and Supplemental Figure 6E). Loss-of-function assays demonstrated that Stattic inhibited the effects of 4-HPA in activating STAT3 and upregulating CXCL3 (Figure 6J and Supplemental Figure 6F). The IF assay showed that 4-HPA promoted the phosphorylation and nuclear translocation of STAT3, whereas Stattic inhibited the phosphorylation of STAT3 induced by 4-HPA (Figure 6, K and L, and Supplemental Figure 6B).

The JAK/STAT3 pathway not only affects the tumor immune microenvironment but is also related to tumor progression directly (29). We speculated that 4-HPA influences tumor growth and viability. EdU cell proliferation experiments showed that 4-HPA promoted the growth of CRC cells in vitro (Supplemental Figure 6G). Furthermore, related metabolites modulate histone deacetylases (HDACs) (30). We explored the effects of 4-HPA on HDACs. The results showed that 4-HPA had a weakly inhibitory effect on HDACs (Supplemental Figure 6H). HDAC loss-of-function and gain-of-function assays showed that HDACs did not affect the stimulating effect of 4-HPA on CXCL3 (Supplemental Figure 6I) or the promoting effect of 4-HPA on CRC proliferation (Supplemental Figure 6J). Therefore, we conclude that 4-HPA does not regulate the CRC phenotypes through HDACs.

Taken together, these results demonstrate that 4-HPA activates JAK2/STAT3 signaling and promotes the transcriptional regulation of CXCL3.

### 4-HPA regulates PMN-MDSC accumulation in CRC and is related to the clinical progression of CRC.

**Figure 7:**
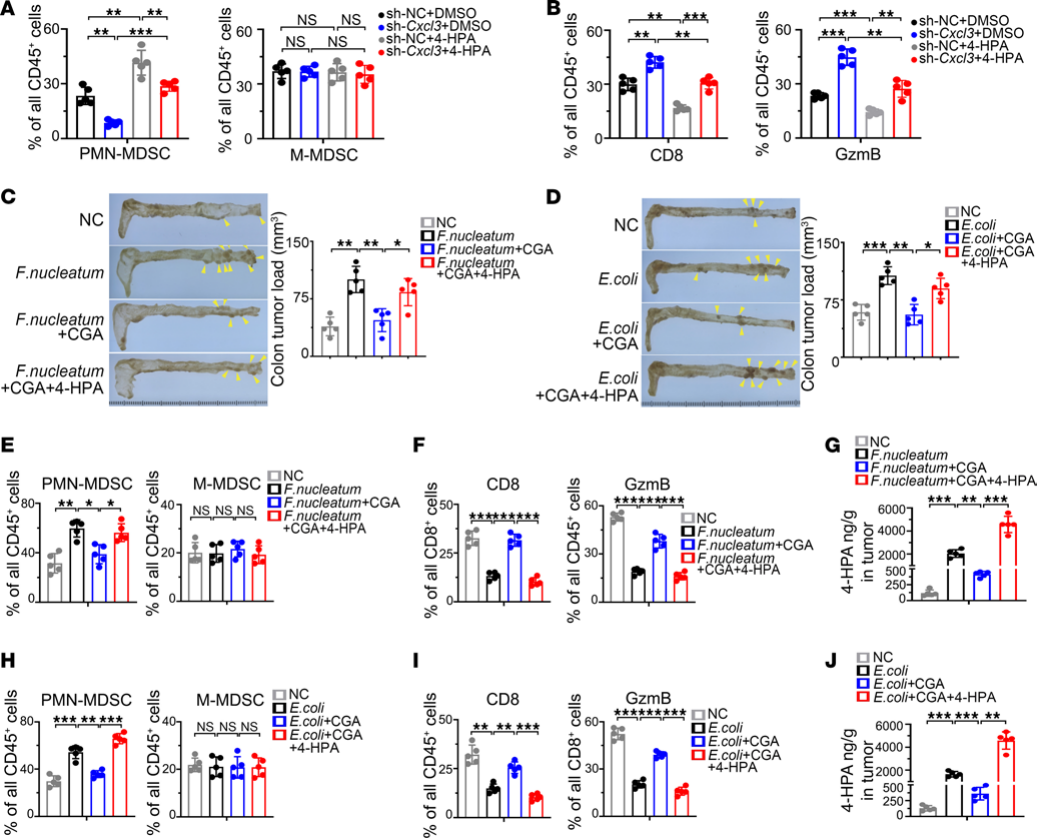
4-HPA correlates with PMN-MDSC accumulation in CRC. (**A** and **B**) Orthotopic implanted CRC mice were fed 4-HPA (1 mM) or control water (DMSO). (**A**) Bar chart of PMN-MDSCs and M-MDSCs measured by flow cytometry (*n* = 5). (**B**) Bar chart of CD8^+^ T cells and GzmB^+^ CD8^+^ T cells measured by flow cytometry (*n* = 5). (**C**–**J**) Results from the Apc^min/+^ tumorigenesis mouse model. (**C**) Tumors in the intestines of Apc^min/+^ mice are shown, as is a bar chart of tumor load of intestines derived from Apc^min/+^ mice colonized with *F. nucleatum* and fed with or without 4-HPA (1 mM) and CGA (1 mM) (*n* = 5). (**D**) Tumors in the intestines of Apc^min/+^ mice are shown, as is a bar chart of tumor load in intestines derived from Apc^min/+^ mice colonized with *E. coli* and fed with or without 4-HPA (1 mM) and CGA (1 mM) (*n* = 5). (**E** and **H**) Bar chart of PMN-MDSCs and M-MDSCs measured by flow cytometry (*n* = 5). (**F** and **I**) Bar chart of CD8^+^ T cells and GzmB^+^ CD8^+^ T cells measured by flow cytometry (*n* = 5). (**G** and **J**) Detection of 4-HPA in tumor tissues of Apc^min/+^ mice by HRGC-MS (*n* = 5). Data represent the mean ± SD of 3 independent experiments. Statistical analyses were conducted using 1-way ANOVA with Dunnett’s T3 correct multiple-comparison test. *P < 0.05, **P < 0.005, ***P < 0.0005. NC, negative control; sh, short hairpin.

Given that 4-HPA stimulates CRC cells to secrete CXCL3, we investigated whether elevated 4-HPA levels in CRC could recruit MDSCs in vivo. Flow cytometry was used to detect MDSCs in the orthotopic implanted CRC mouse model, consistent with previous research, in which tumors of mice fed 4-HPA contained a markedly higher abundance of PMN-MDSC cells, and knockdown of *Cxcl3* eliminated the promoting effect of 4-HPA on PMN-MDSC cells. However, changes in M-MDSC were not statistically significant (Figure 7A). Flow cytometry also revealed a marked decrease of GzmB^+^ CD8^+^ T cells in tumors of mice fed 4-HPA (Figure 7B).

Chlorogenic acid (CGA), which has antibacterial effects (31), reduces the production of 4-HPA by inhibiting the shikimic acid pathway (32). We used the Apc^min/+^ mice tumorigenesis model to explore whether CGA can reverse the immunosuppressive microenvironment. The results revealed that CGA reduced the tumor burden of Apc^min/+^ mice and reduced the severity of tumor-related lesions (Figure 7, C and D, and Supplemental Figure 7, A–D). Additional flow cytometry studies demonstrated that CGA inhibited the chemotactic effect of *F. nucleatum* on PMN-MDSCs, and this inhibitory effect was blocked by 4-HPA in Apc^min/+^ mice (Figure 7E). Simultaneously, CGA eliminates the inhibitory effect of *F. nucleatum* on GzmB^+^ CD8^+^ T-cell infiltration, and this effect was blocked by 4-HPA in Apc^min/+^ mice(Figure 7F). A similar phenomenon was observed in mice treated with *E. coli* (Figure 7, H and I). High-resolution gas chromatography–mass spectroscopy (HRGC-MS) was used to measured 4-HPA in Apc^min/+^ mice and showed that CGA reduced the content of microbial metabolite 4-HPA in vivo (Figure 7, G and J).

**Figure 8:**
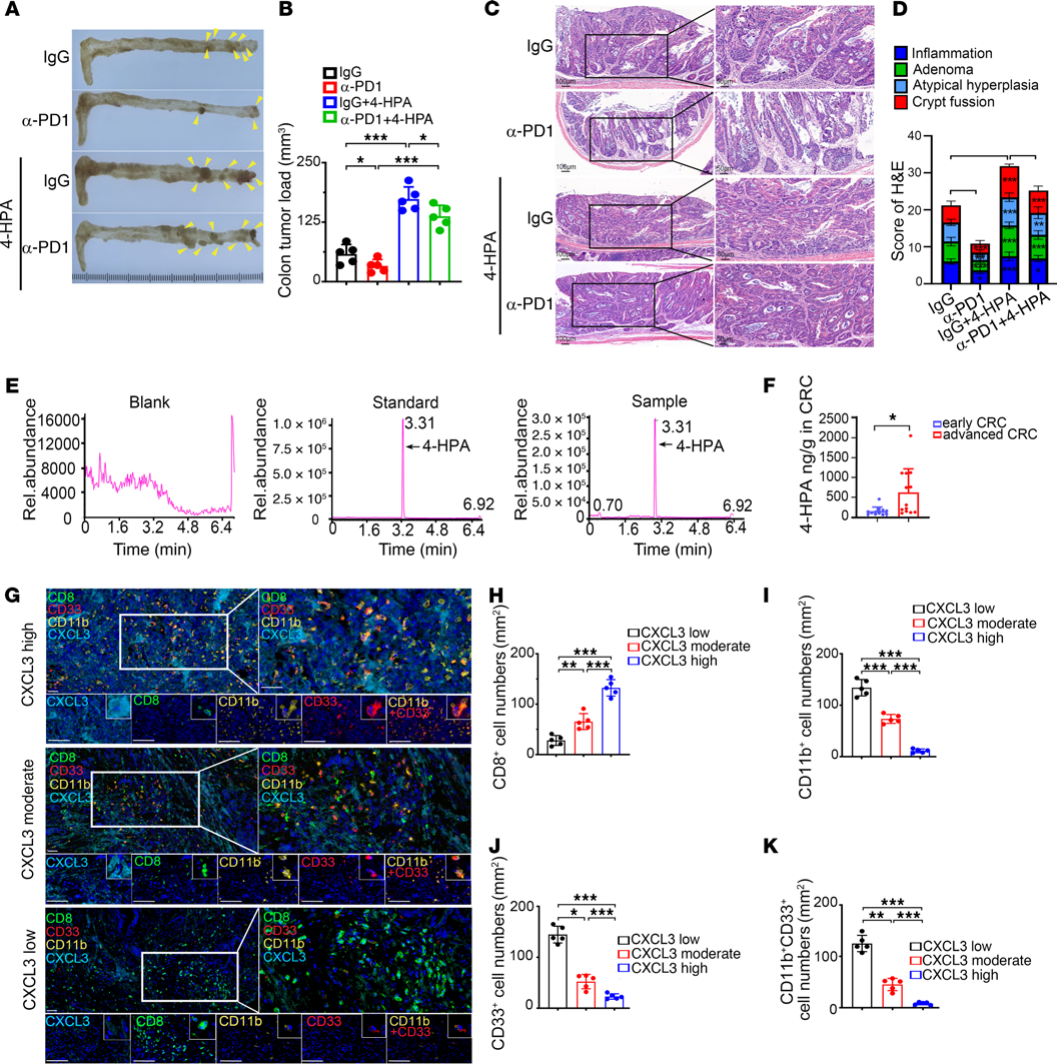
4-HPA correlates with PMN-MDSC accumulation in CRC. (**A**) Tumors in the intestines of Apc^min/+^ tumorigenesis mice (*n* = 5). (**B**). Bar chart of tumor load in intestines derived from Apc^min/+^ mice treated with PD-1 immunotherapy or IgG, with or without 4-HPA (1 mM) (*n* = 5). (**C**) Representative images of intestines tumorigenesis visualized by H&E staining. (**D**) H&E scoring of tumor-related lesions (including inflammation, adenoma, atypical hyperplasia, and crypt fusion) (*n* = 5). (**E** and **F**) Detection of 4-HPA in tumor tissues of patients with CRC by HRGC-MS (*n* = 12). (**G**–**K**) Expression of CD8, CD33, CD11b, and CXCL3 in CRC tissues of patients with CRC analyzed by multiple IF. Visualization of 3 representative cases is shown. Scale bar: 50 μm. Multiple IF detection was performed on the tumor tissues of patients with CRC (**G**). Bar charts of CD8^+^ T cells (**H**), CD11b^+^ cells (**I**), CD33^+^ cells (**J**), and MDSCs (CD11b^+^CD33^+^) (**K**) in CRC tissues (*n* = 5). Data represent the mean ± SD of 3 independent experiments. We used 2-way ANOVA to determine the significance of tumor volume of PD-1 treated mice and H&E staining. The remaining statistical analyses were conducted with Student’s *t* test (2-comparison test) and 1-way ANOVA with Dunnett’s T3 correct multiple-comparison test. *P < 0.05, **P < 0.005, ***P < 0.0005. rel., relative.

Furthermore, we constructed an Apc^min/+^ tumorigenesis mouse model to verify the effect of 4-HPA on the efficacy of PD-1 immunotherapy in mice. The results showed that 4-HPA blocked the efficacy of PD-1 antibodies and promoted tumorigenesis in Apc^min/+^ mice (Figure 8, A–D). HRGC-MS analysis of patients with clinical CRC revealed that 4-HPA levels were substantially higher in patients with advanced CRC (Figure 8, E and F). Additionally, multiple IF of clinical CRC samples further validated that CXCL3 was negatively correlated with CD8^+^ T cells but positively correlated with CD33^+^ CD11b^+^ MDSC cells in tumors (Figure 8, G–K). IHC staining confirmed the expression of CD8, CD3, CD11b, and CXCL3 in clinical CRC samples (Supplemental Figure 8, A and B).

In vivo, compared with untreated mice, mice treated with 4-HPA had more advanced CRC progression, characterized by larger tumors (Supplemental Figure 8, C and D) and increased liver metastases (Supplemental Figure 8, E and F). Collectively, these results suggest that 4-HPA promotes the formation of an immunosuppressive microenvironment in CRC and is associated with the clinical progression of CRC.

## Discussion

Intestinal tumors are constantly exposed to intestinal microbiota and microbial metabolites. Early studies often regarded commensal microbiota as pathogens and implicated them as a cause of CRC (33). However, recent study has revealed that the composition of the microbiota is a crucial factor in regulating antitumor immunity and determining the efficacy of ICB therapy. The intestinal microbiota affects the antitumor efficacy of immunotherapy through both congenital and adaptive immunity (34, 35). Moreover, modulating the intestinal microbiome enhances therapeutic responses (36, 37). Despite these insights, research on the consistency and underlying mechanisms linking specific bacteria to immunotherapy outcomes remains limited. As a result, the application of therapies aimed at improving the intestinal microecology in clinical CRC treatment is currently restricted. Given these gaps, there is an urgent need to further elucidate the influence and mechanism of specific intestinal microbiota in immunotherapy.

Here, we investigated 2 pathobiont bacteria closely associated with CRC: *E*. *coli* and *F. nucleatum*. We found that both *E. coli* and *F. nucleatum* stimulated CRC cells to secrete CXCL3 and CCL20 in vitro. We compared the effects of CXCL3 and CCL20 on tumor progression and found that knocking down either CXCL3 or CCL20 substantially inhibited tumor progression in both Balb/C mice and nude mice. However, in Balb/C mice, CXCL3 demonstrated a stronger tumor inhibitory effect than did CCL20. CCL20 is a chemokine that can interact with multiple cytokines and Toll-like receptors, resulting in increased tumor aggression. CCL20-CCR6 exerts its biological effects through regulation of several signaling pathways, including the ERK and NF-κB pathways, as well as the epithelial-mesenchymal transition (38–40). Similarly, CXCL3 plays an important role in various human cancers by regulating the differentiation, invasion, and migration of tumor cells. Studies have shown that CXCL3 promotes tumor progression by activating signaling pathways such as ERK/MAPK in the TME (41). Our study suggested that inhibiting CCL20 or CXCL3 can directly inhibit tumor progression. However, the impact of CCL20 on the tumor immune microenvironment is weaker than that of CXCL3. Given the important role of CXCL3 in promoting the formation of an immunosuppressive microenvironment, our research focused on exploring the impact of metabolites on the tumor immune microenvironment, with a particular emphasis on the cytokine CXCL3.

Furthermore, we confirmed that CXCL3 knockdown inhibited CRC progression by reducing the recruitment of PMN-MDSCs in vivo. There are 2 different types of MDSC: PMN-MDSC and M-MDSCs (42). M-MDSCs are mainly chemotactic through the binding of the CCR2 receptor with chemokines such as CCL2, CCL8, and CCL12, and they also play a variety of immunosuppressive and tumor-promoting roles in the TME. These roles include suppressing the activity of T cells and NK cells by secreting immunosuppressive cytokines (e.g., TGF-β, IL-10) and producing metabolites (e.g., arginase, nitric oxide) (43). Our observations suggest CXCL3 is a major chemotactic factor affecting the tumor immune microenvironment in mouse models of imbalanced microbiota and can attract PMN-MDSCs that express CXCR2 receptor, rather than M-MDSCs.

Although recent studies have indicated an association between commensal bacteria and the TME, much of the existing research is associative (44). There are still many mechanisms that have not been decoded. Intestinal microbiota metabolize many small-molecule metabolites, and these products have completely different biological functions, revealing innovative causal relationships and mechanisms between microbiota metabolites and the tumor immune microenvironment may help improve the efficacy of CRC immunotherapy. In this study, we found that the microbial metabolite 4-HPA substantially stimulates the secretion of CXCL3 in CRC cells, controls chemokine-dependent accumulation of PMN-MDSCs, and restrains antitumor immunity in CRC. Further experiments with FITC-labeled 4-HPA revealed that 4-HPA localized in the cytoplasm and nucleus of cells and increased the phosphorylation level of the transcription factor STAT3. Phosphorylated STAT3 (p-STAT3) binds to the CXCL3 promoter and regulates its transcription. Additionally, we observed notably elevated levels of 4-HPA in patients with advanced CRC, which emphasizes its clinical relevance. To date, few studies have explored the specific mechanisms by which microbial metabolites participate in the tumor immune microenvironment. Specific blocking of immunosuppressive factors in the TME is expected to improve the antitumor immune response. Given that a positive response to immunotherapy relies on the immunomodulatory interaction between tumor cells and the TME, identifying and elucidating the immunosuppressive factors in the TME are expected to provide a new method to improve the effectiveness of ICB therapy.

### Conclusion.

**Figure 9:**
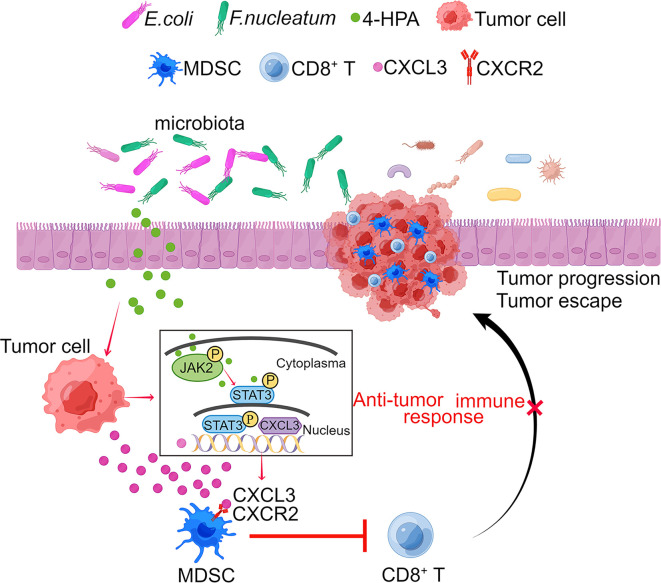
A schematic of the microbial metabolite 4-HPA mediating CRC immunosuppression. The gut microbiome uses 4-HPA as a messenger to regulate chemokine CXCL3 level in CRC cells, thereby controlling the accumulation of CXCR2^+^ PMN-MDSCs. The accumulated PMN-MDSCs inhibit the antitumor effect of CD8^+^ T cells.

In summary, in this study, we identified pathobiont bacteria and their metabolites as potential therapeutic targets for modulating the antitumor response in CRC. We revealed a mechanism by which the gut microbiota uses 4-HPA as a messenger to control the chemokine-dependent accumulation of PMN-MDSCs and regulate antitumor immune response in CRC (Figure 9). Our metabolomics-based research provides compelling evidence of the role of microbiota in tumor immunity, underscoring the potential for future therapeutic interventions targeting gut microbiota and their metabolites.

## Methods

Supplemental Methods are available online with this article.

### Sex as a biological variable.

Our study examined male and female patients and animals, and similar findings are reported for both sexes.

### Cell culture.

CRC cell lines SW480, HCT116, RKO, and the mouse colon carcinoma cell line CT26 were obtained from the Cell Bank of the Chinese Academy of Sciences and maintained as previously described (27). All cells were cultured in RPMI-1640 medium (HyClone) supplemented with 10% FBS (ExCell Bio) at 37°C and 5% CO_2_.

### Mouse models and animal treatment.

Apc^min/+^ mice (C001196) were purchased from Cyagen. BALB/c mice (aged 4–6 weeks) and nude mice (aged 4–6 weeks), obtained from Nanfang Medical University Experimental Animal Center, were housed in a specific pathogen-free animal room and had free access to sterilized food and water. The experimental scheme has been described previously (27). In each experiment, the mice were age- and sex-matched and randomly assigned to different experimental groups. For the in vivo experiments related to animals, there were 5 biological replicates.

For the Apc^min/+^ tumorigenesis mouse model, Apc^min/+^ mice at 6 weeks old were i.p. injected with a single dose of AOM (10 mg/kg; Sigma-Aldrich), followed by 2 cycles of 7-day supplementation of 2% dextran sulfate (DSS; MP Biomedicals) in drinking water. Each cycle was separated by a 14-day resting period without DSS administration (Supplemental Figure 1B). Apc^min/+^ mice were sacrificed 9 weeks after treatment. Paraffin sections of large intestines were prepared and stained with H&E. According to the blinding and randomization method, 2 pathologists determined the degree and number of tumor-related lesions in each sample, and scored them for 4 dimensions: inflammation, adenoma, atypical hyperplasia, and crypt fusion, with scoring ranging from 0 (none) to 10 (severe).

For the orthotopic implanted CRC mouse model, CT26 cells were washed with PBS and filtered through a 40 μm strainer. Before tumor cells were inoculated, age- and sex-matched mice (aged 6–8 weeks) were anesthetized and shaved first, then 1 × 10^6^ CT26 cells were injected underneath the intestinal serosa. The colorectum and liver were dissected and stained with H&E for pathological observation of tumor lesions. The tumor nodules were counted under ×10 low power microscope, the number of tumor nodules was recorded and analyzed. This study was approved and performed in accordance with relevant guidelines of Southern Medical University.

For imbalance of the gut microbiota mouse model, Apc^min/+^ mice or BALB/c mice were randomized into 5 mice/cage and housed for 1 week to normalize the gut microbiome. Then mice were assigned into bacterial strain (*E. coli or F. nucleatum*) or PBS treatment groups. Gut microbiota was removed by intragastric antibiotics treatment (a mixture of neomycin 200 mg/kg, penicillin 200 mg/kg, metronidazole 200 mg/kg, and vancomycin 100 mg/kg) for 5 days to deplete the gut microbiota (36). After the antibiotic treatment, the mice were orally administrated 1 × 10^8^ CFU *F. nucleatum*, *E*. *coli*, or PBS daily for 3 days, then twice a week for maintaining microbiota colonization. Metagenomic sequencing of mice feces was applied to confirm the successfully establishment of the animal model. The mice were sacrificed and tumors were harvested. For survival analysis, mice were sacrificed when moribund, and survival were analyzed with a Kaplan-Meier curve.

For in vivo anti–PD-1 treatment, PD-1 blockade was performed with anti–PD-1 monoclonal antibody (BioXCell, BE0146, clone RMP1-14) and IgG isotype control (BioXCell, BE0089, clone 2A3), given 3 times a week through i.p. injections at a dose of 200 μg/injection. Cytokine neutralization was performed with anti-*Cxcl3* monoclonal antibody (sheep; R&D Systems, AF5568), anti-*Ccl20* monoclonal antibody (rabbit; R&D Systems, MAB7601), and IgG isotype control (BioXCell, BE0090, clone 2A3) given by i.p. injection twice a week at a dose of 200 μg/injection.

For the construction of CRC xenograft model, 5 × 10^5^ cells administered via subcutaneous inoculation in each mouse. After 4 weeks, mice were sacrificed and the xenograft tumors were quickly harvested for histologic study. The tumor volume was calculated according to the following formula: volume (mm^3^) = width^2^ (mm^2^) × length (mm)/2.

For CT26 tumor-bearing mice, the mice were treated with 4-HPA (1 mM; S31340, Yuanye Bio-Technology) in daily drinking water. To investigate effect of CGA, the mice were orally administered CGA (1 mM; S30617, Yuanye Bio-Technology) in daily drinking water (45).

### Bacterial strains and growth conditions.

*E. coli* bacterial strains were human fecal-isolate strains obtained from patients with CRC in Nanfang Hospital and were identified as polyketide synthase–positive (pks+) *E. coli* and stored in our laboratory. Bacterial inocula were cultured in Luria-Bertani broth overnight at 37°C with shaking at 220 rpm to the mid-log phase. *F. nucleatum* bacterial strains (ATCC, 25586) were obtained from the Guangdong Microbial Culture Collection Center (46). After adjusting the Center’s culture recommendations, *F. nucleatum* was retrieved from freeze-dried powder (10% skimmed milk, 1.5% fucose, 0.5% glycerol, 2% sorbitol, 1% maltodextrin). Bacteria were cultured overnight in brain heart infusion broth supplemented with hemin, K_2_HPO_4_, vitamin K_1_, and l-cysteine and maintained under anaerobic conditions (DG250, Don Whitley Scientific) at 37°C, 95% N_2_, and 5% CO_2_(46). Bacteria strains were identified by Beijing Liuhe BGI Technology Co., Ltd. Bacterial genomic DNA was extracted and the 16S bacterial conservative sequence was amplified by PCR using 3730 sequencing; then, sequencing results were compared with the NCBI database using BLAST. The blast results showed that *F. nucleatum* culture was closest to *F. nucleatum* subsp. *nucleatum* ATCC 25586 and *E. coli* culture was closest to *E. coli* ATCC 25922. The bacterial count was confirmed to be 1×10^10^ CFU/g.

### Co-incubation of bacterial strains and CRC cells.

The concentration of bacteria was adjusted based on the optical density reading at 600 nm, as analyzed using a NanoDrop ND-2000 spectrophotometer. Bacteria were diluted to 1:10 (*E. coli*) (27) or 1:100 (*F. nucleatum*) (47, 48) with the appropriate medium prior to co-culture with CRC cells. Briefly, CRC cells were grown to 70% confluence before being co-cultured with the bacterial strains in RPMI-1640 medium supplemented with 10% FBS for 6 hours. Bacterial strains were allowed to infect monolayers of CRC cells. The cell culture supernatant was collected and bacteria were removed by filtration using a 0.22 μm membrane. All extracellular bacteria that infected CRC cells were killed by gentamicin (500 μg/mL for 20 minutes), and dead bacteria were removed by extensive washing with PBS (27). Culturing of CRC cells was continued using the collected cell culture supernatant for 48 hours.

### Metagenomic sequencing.

Total fecal DNA was extracted using the MagPure Stool DNA KF kit B (Magen) and sequenced on the Illumina NovaSeq 6000 platform with two 150 bp paired-end reads. DNA libraries were prepared and subjected for taxonomic profiling using the NEB Next Ultra II FS DNA library prep kit for Illumina (E6177, New England Biolabs). DNA (1 ng) was subjected to fragmentation enzyme for 10 minutes at 37^o^C, followed by adaptor ligation. The adaptor was diluted 25-fold, and 2.5 μL of the diluted adaptor was incubated with 35 μL of fragmented DNA, 30 μL of NEB Next Ultra II ligation master mix, and 1 μL of NEB Next ligation enhancer at 20°C for 15 minutes. We added 3 μL of USER enzyme to the ligation mixture and incubated the mixture at 37°C for 15 minutes. Purification with beads was carried out according to the manufacturer’s instructions prior to PCR enrichment of adaptor-ligated DNA under the following conditions: an initial temperature of 98°C for 30 seconds, followed by 12 cycles of 98°C for 10 seconds, 65°C for 75 seconds, then 65°C for 5 minutes.

### Cytokine antibody array.

Human Cytokine Array I (RayBio Human Cytokine Antibody Array, AAH-CYT-G5) detects 80 human cytokines and chemokines. The glass chip was placed in the laminar flow hood to completely dry for 1–2 hours according to the manufacturer’s instructions. Then 100 μL of 1× blocking buffer was added to each well and incubated at room temperature (RT) for 30 minutes to block slides. The blocking buffer then was removed and 80 μL of each sample was added to each subarray. The arrays were incubated with the samples at 4°C overnight. A Thermo Scientific Wellwash Versa microplate washer was used to clean the slides. The slides were washed 10 times for 10 seconds each with 250 μL of 1× Wash Buffer I at RT. The entire glass chip assembly was submerged and washed for 10 minutes at RT with gentle rocking or shaking, then it was washed 6 times for 10 seconds each with 250 μL of 1× Wash Buffer II at RT. The entire glass chip assembly was submerged and washed for 10 minutes at RT with gentle rocking or shaking. We added 70 μL of 1× biotin-conjugated anti-cytokines to each subarray, and this was incubated at RT for 2 h with gentle rocking or shaking, then washed as described before. We then added 70 μL of 1× Streptavidin-Fluor to each subarray, and then either the entire assembly was covered with aluminum foil to avoid exposure to light or it was incubated in a dark room at RT for 2 hours with gentle rocking or shaking. The assembly was washed as described previously. The glass chip was scanned with a laser scanner (e.g., Innopsys’ InnoScan) using the cy3 or “green” channel (excitation frequency, 532 nm). The measured cytokine panels are listed in Supplemental Table 1.

### Isolation of TILs and flow cytometric analysis.

The dissociation buffer consisted of 2 mg/mL collagenase type IV (Thermo Fisher Scientific), 0.4 mg/mL hyaluronidase (MilliporeSigma), 0.5% Pen-Strep Solution (Biological Industries [BI]; containing 10,000 U/mL penicillin G sodium salt and 10 mg/mL streptomycin sulfate), and 100 U/mL DNase (MilliporeSigma) in RPMI medium with 10% heat-inactivated FCS. The tumor was cut with autoclaved, sterile surgical scissors and yielded a mixture of cells. The tumor was then digested with dissociation buffer at 37°C for 1 hour. Samples were filtered through a 70 μm cell filter and then subjected to Percoll (Yeasen, 40501ES76) density gradient centrifugation. TILs were separated by centrifuging at 1,000*g* and 4°C for 20 minutes; the dead cells were centrifuged to the bottom of the tube, and the transparent layer between Percoll and the medium was collected. Trypan blue staining was used to identify the living cells.

For cell surface staining, cells were incubated with Fc Block (BD Pharmingen, 553142) at RT for 10 minutes and then stained with antibodies against surface antigens at 4°C for 30 minutes, including those against CD45 (rabbit; Thermo Fisher, 25-0451-81; PE-CYN7, 1:400), CD11b (rabbit; BioLegend, 101212; APC, 1:200), Ly-6C (rabbit; BioLegend, 128011; PerCP/Cyanine5.5, 1:200), Ly-6G (rabbit; BioLegend, 127607; phycoerythrin [PE], 1:200), CD3 (rabbit; BioLegend, 100217; PerCP/Cyanine5.5, 1:200), CD4 (rabbit; BioLegend, 100509; FITC, 1:200), and CD8a (rabbit; BioLegend, 100711; APC, 1:100). All stains were performed in flow cytometry wash buffer (PBS containing 1% FBS and 0.5 mM EDTA) at 4°C for 30 minutes. For intracellular cytokines, cells were stimulated with leukocyte activation cocktail with BD GolgiPlug (BD Pharmingen, 550583) for 4 hours and stained with intracellular antibodies, including those against FOXP3 (mouse; BioLegend, 320007; PE, 1:200), granzyme B (mouse; BioLegend, 372215; PE/Dazzle594, 1:500), TNF-α (rabbit; Thermo Fisher, 12-7321-81; PE, 1:200), and IFN-γ (rabbit; Thermo Fisher, 48-7311-82; EF450, 1:200). Flow cytometry was performed on an LSRFortessa cell analyzer (BD Biosciences), and data were analyzed using FlowJo (Treestar).

### MDSC isolation and in vitro migration assay.

MDSCs were isolated from the spleens of C57/B6 mice using a mouse MDSC Isolation Kit (Miltenyi Biotec, 130-094-538) and plated in RPMI-1640 medium supplemented with 10% FBS and antibiotics. MDSCs (1 × 10^5^ cells/well) were seeded in the top chamber of the transwell. Conditioned media (CM) from cultured CRC cell lines were collected and added to the bottom layer of the transwell. We generated 3 types of migration assay during our study: (a) for the CXCL3 chemotaxis assay, *Cxcl3* CM were from mice CT26 cell lines, which was subjected to *Cxcl3* overexpression for 48 hours; (b) for the microbiota chemotaxis assay, *E. coli/F. nucleatum* CM were from mice CT26 cell lines, which were subjected to co-incubation with *E. coli* or *F. nucleatum* and CT26 cells for 48 hours; and (c) for the CXCL3/CXCR2 axis assay, α-*Cxcl3* CM were from mice CT26 cell lines added to CXCL3 neutralizing antibody (mouse; R&D, AF5568) or CXCR2 neutralizing antibody (rabbit; R&D, MAB2164) in MDSCs. After a 6-hour incubation, MDSCs that had completely migrated to the bottom chamber were counted.

### Construction of FITC-labeled 4-HPA.

FITC-labeled 4-HPA was constructed by Ruixi Biotech. We dissolved 20 mg of 4-HPA in 2 mL of N,N-dimethylformamide (DMF) and added 1-Ethyl-3-(3-dimethylaminopropyl) carbodiimide (EDC; 3.0 equivalent [eq.]) and N-Hydroxysuccinimide (NHS; 3.0 eq.). The DMF solution was completely dissolved, stirred, and reacted for 4 hours at RT, purified by column chromatography, and dried under vacuum to obtain the 4-HPA-NHS product. Then 10 mg of 4-HPA–NHS was dissolved in 1 mL of DMF and amino fluorescein (1.2 eq.) was added. The DMF solution was completely dissolved, stirred, and reacted for 0.5 hours at RT, purified by column chromatography, and dried under vacuum to obtain the FITC-labeled 4-HPA.

### 4-HPA analysis.

HRGC-MS was used to detect 4-HPA content. The standard is used as a reference to accurately quantify 4-HPA in the experimental sample: the ions are screened by the quadrupole to obtain the parent ion (Q1), only the selected parent ion is broken up at a specific collision energy, the specific daughter ion (Q3) is obtained by the quadrupole screening, and the mass spectrum signal is collected

### WB analysis.

CRC cells were treated with 4-HPA (1 mM). The main chemical reagents used in WB were Stattic (Selleckchem, S7024; 20 μM), Colivelin (Selleckchem, S9664; 20 μM), ITSA-1 (Selleckchem, S8323; 50 μM), and Panobinostat (Selleckchem, S1030; 50 nM). Immunoblot analysis of cell lysates (20–60 mg) in RIPA buffer was carried out to assess protein expression in the presence of rabbit antibodies against IRF1 (rabbit; Proteintech, 11335-1-AP; 1:1,000), OCT1 (rabbit; Proteintech, 10387-1-AP; 1:1,000), HOXD9 (rabbit; Proteintech, 20560-1-AP; 1:1,000), HOXD10 (rabbit; Abcam, ab138508; 1:1,000), p-STAT3 (rabbit; CST, 9145; 1:1,000), STAT3 (mouse; CST, 9139; 1:1,000), JAK2 (rabbit; CST, 3230; 1:1,000), p-JAK2 (rabbit; CST, 3771; 1:1,000), CXCL3 (rabbit; Thermo Fisher Scientific, PA5-103136; 1:1,000), and GAPDH (mouse; ZSbio, TA-08; 1:1,000).

### IF assays.

IF staining was performed according to the standard protocol described previously (49). A polyclonal primary rabbit antibody CXCR2 (rabbit; R&D Systems, MAB2164; 1:200), PE anti–mouse Gr-1 antibody (rabbit; Multi Sciences, F21LY6G02; 1:200), p-STAT3 (rabbit; CST, 9145; 1:200), and STAT3 (rabbit; Affinity, AF6294; 1:200). DAPI was also used in the experiment.

For multiple IF, as shown in Figure 6C, human intestinal tissues from patients were placed in 4% paraformaldehyde for 24 hours, dehydrated, embedded in paraffin, and sectioned into 5 μm slices for use. Sections were stained using an IF kit (G1236-100T, Servicebio) according to the manufacturer’s instructions. The image data were calculated using Aipathwell digital pathology image analysis software (Servicebio). The following antibodies and corresponding fluorescent dyes were used for multiplex immunofluorescence staining: CD8 (rabbit; Abcam, ab237709; 1:100), CD33 (rabbit; Abcam, ab269456; 1:200), CD11b (rabbit, Abcam, ab52478; 1:200), and CXCL3 (rabbit; Immunoway, YT2075; 1:200).

### IHC assays.

IHC was performed to investigate the expression of proteins in human CRC tissues, according to the protocol described previously (27). The sections were incubated overnight using primary antibodies CD8 (rabbit; Abcam, ab209775; 1:1,000), CD4 (rabbit; Bioss, bs-0647R; 1:1,000), FOXP3 (rabbit; eBioscience, 14-4771-80; 1:300), Gr-1 (rabbit; BioLegend, 108401; 1:300), CXCL3 (rabbit; Immunoway, YT2075; 1:1,000), CD8 (rabbit; Zsbio, ZA-0508), CD33 (rabbit; Abcam, ab269456), and CD11b (mouse; ORIGENE, TA807952; 1:500) at 4°C. An HRP-conjugated secondary antibody and DAB staining kit (CWBIO) were used in the experiment.

### Statistics.

Statistical analyses were carried out using GraphPad Prism 9 (GraphPad Software Inc.). All values are expressed as the mean ± SD. All the experiments were independently repeated at least 3 times, but only the representative figure is displayed here. For normally distributed data, an unpaired, 2-tailed Student’s *t* test was used for 2-group comparisons, and 1-way ANOVA with Dunnett’s T3 tests was used for multiple group comparisons. For tumor growth assays, we used 2-way ANOVA followed by Tukey’s multiple comparisons test. For the survival curves, pairwise comparisons were performed using the Mantel-Cox test to compute *P* values. These *P* values were then adjusted using the Benjamini-Hochberg function in R (version 4.4.0). A *P* value less than 0.05 was considered significant.

### Study approval.

All experiments involving patients were approved by the Ethics Committee of Shunde Hospital, Southern Medical University (approval KYLS20230918) and complied with the Declaration of Helsinki. Informed consent was not required, because the data were analyzed anonymously. All animal experiments were performed according to the ethical guidelines approved by Southern Medical University Animal Care and Use Committee (approval NFYY-2019-0921).

### Data availability.

All source data values are provided in the Supporting Data Values file. All other underlying data and any supporting analytic code in this article are available from the corresponding author upon request. Additional methods are provided in the supplemental material.

## Author contributions

LZ, YD, and HW designed the study and prepared the manuscript. QL, X Zhou, LW, and YY performed experiments. X Zhu performed the statistical analyses. HL and YZ assisted with tissue sample collection. WL and FZ performed the data analysis and interpretation. All authors approved the final version of the manuscript.

## Supplementary Material

Supplemental data

Unedited blot and gel images

Supporting data values

## Version 1 04/03/2025

In-Press Preview

## Version 2 06/02/2025

Electronic publication
