# Corrigendum to Gut microbial metabolite 4-hydroxybenzeneacetic acid drives colorectal cancer progression via accumulation of immunosuppressive PMN-MDSCs

**DOI:** 10.1172/JCI199353

**Published:** 2025-10-01

**Authors:** Qing Liao, Ximing Zhou, Ling Wu, Yuyi Yang, Xiaohui Zhu, Hangyu Liao, Yujie Zhang, Weidong Lian, Feifei Zhang, Hui Wang, Yanqing Ding, Liang Zhao

Original citation: *J Clin Invest*. 2025;135(11):e181243. https://doi.org/10.1172/JCI181243

Citation for this corrigendum: *J Clin Invest*. 2025;135(19):e199353. https://doi.org/10.1172/JCI199353

The original article omitted the following information for accessing sequencing data:

The raw sequencing data were deposited in a public repository, the NCBI Sequence Read Archive (SRA) (https://www.ncbi.nlm.nih.gov/sra; Bioproject accession number PRJNA1302563). Group-level metadata are available from the corresponding author under execution of a streamlined Data Transfer Agreement (DTA) upon reasonable request and IRB approval.

The HTML and PDF files have been updated with this information.

The authors regret the error.

## Version 1 10/01/2025

Electronic publication

