# Supplementary material for: Gut microbial metabolite 4-hydroxybenzeneacetic acid drives colorectal cancer progression via accumulation of immunosuppressive PMN-MDSCs
Source: J Clin Invest. 2025 Apr 3;135(11):e181243. doi: 10.1172/JCI181243 (PMC12126219; doi:10.1172/JCI181243)
Supplement: Supplemental data [file jci-135-181243-s040.pdf]

Supplementary Materials for

Gut microbial metabolite 4-hydroxybenzeneacetic acid drives colorectal cancer progression via accumulation of immunosuppressive PMN-MDSCs

Qing Liao*, Ximing Zhou*, Ling Wu* Yuyi Yang*, Xiaohui Zhu, Hangyu Liao, Yujie Zhang, Weidong Lian, Hui Wang[&], Yanqing Ding[&], Liang Zhao[&]

Correspondence to: Liang Zhao (liangsmu@foxmail.com), Yanqing Ding (dyqgz@126.com) or Hui Wang (wanghui_0226@126.com)

This PDF file includes:

Supplementary Materials and Methods

Supplementary Figures 1 to 8

Supplementary Tables 1 to 4

Supplementary Materials and Methods

Metabolomic analysis

200 mg accurately weighed mice colorectal contents sample was transferred to a 1.5-mL Eppendorf tube. Two small steel balls were added to the tube. 20 μL internal standard (2-chloro-l-phenylalanine in methanol, 0.3 mg/mL) and 600 μL extraction solvent with methanol /water (4/1, v/v) were added to each sample. Samples were stored at -80ºC for 2 min and then grinded at 60 HZ for 2 min. 120 μL of chloroform was added to the samples, then the samples were vigorously vortexed and followed by 10 min ultrasound-associated extraction at ambient temperature, then stored at 4ºC for 10 min. The samples were centrifuged at 12000 rpm for 10 min at 4ºC. QC sample was prepared by mixing aliquots of the all samples to be a pooled sample. An aliquot of the supernatant was transferred to a glass sampling vial for vacuum-dry at room temperature. And 80 μL of methoxylamine hydrochloride (dissolved in pyridine, 15 mg/mL) was subsequently added. The resultant mixture was vortexed vigorously for 2min and incubated at 37ºC for 90 min. 80 μL of BSTFA (with 1% TMCS) and 20 μL n-hexane were added into the mixture, which was vortexed vigorously for 2 min and then derivatized at 70ºC for 60 min. The samples were placed at ambient temperature for 30 min before GC-MS analysis. The derivatived samples were analyzed on an Agilent 7890B gas chromatography system coupled to an Agilent 5977A MSD system (Agilent Technologies Inc., CA, USA). A DB-5MS fused-silica capillary column (30 m × 0.25 mm × 0.25 μm, Agilent J & W Scientific, Folsom, CA, USA) was utilized to separate the derivatives. Helium (>99.999%) was used as the carrier gas at a constant flow rate of 1 mL/min through the column. The injector temperature was

maintained at 260 ºC. The initial oven temperature was 60 ºC, ramped to 125 ºC at a rate of 8 ºC/min, to 210 ºC at a rate of 5 ºC/min， to 270 ºC at a rate of 10ºC/min, to 305 ºC at a rate of 20 ºC/min, and finally held at 305 ºC for 5 min. The temperature of MS quadrupole and ion source (electron impact) was set to 150 and 230 ºC, respectively. The collision energy was 70eV. Mass spectrometric data was acquired in a full-scan mode (m/z 50-500). The QCs were injected at regular intervals throughout the analytical run to provide a set of data from which repeatability could be assessed.

Analysis Base File Converter software was used to convert the raw data (.D format) to .abf format, and then the .abf data were imported into the MD-DIAL software for data processing. Metabolites were annotated through LUG database (Untarget database of GC-MS from Lumingbio). In this study, the default 7-round cross-validation was applied with 1/seventh of the samples being excluded from the mathematical model in each round, in order to guard against overfitting. The differential metabolites were selected on the basis of the combination of a statistically significant threshold of variable influence on projection (VIP) values obtained from the OPLS-DA model and p values from a two-tailed Student's t-test on the normalized peak areas from different groups, where metabolites with VIP values larger than 1.0 and p values less than 0.05 were considered as differential metabolites.

Clinical samples

For metagenomic sequencing, faecal samples were collected from 20 CRC patients and 20 Age-, sex-, and BMI-matched healthy volunteers of Southern Hospital (2024) and stored at -80 ºC until DNA extraction. For 4-HPA HRGC-MS test sample, a total of 12 tumor tissue

samples from patients with early CRC, and 12 tumor tissue samples from patients with advanced CRC were obtained from the tumor tissue bank of Southern Hospital. Each CRC patient was diagnosed with primary CRC. All the patients did not receive radiotherapy and chemotherapy before operation and there was no history of other malignant tumors, and the patient had undergone elective surgery for CRC in Nanfang Hospital between (2018 and 2024). The tumor clinical stage information was extracted from the case analysis. Early CRC defined as primary tumor was confined to the intramucosal or submucosa, without lymph node metastasis or distant metastasis. Advanced CRC defined as primary tumor had invaded the muscular layer but no lymph node metastasis or distant metastasis was present, or regardless of the depth and extent of primary tumor invasion, regional lymph node metastasis or distant metastasis was present. All the samples were randomly selected, except for the integrity of patient follow-up data may lead to the emergence of potential self-selection bias, other factors will not lead to self-selection bias. The study was approved by the Ethics Committee of Southern Medical University and all aspects of the study are in line with the Declaration of Helsinki. No informed consent was required because data were analyzed anonymously.

Metabolites Treatment

Differential metabolites purchased from Yuanye Bio-Technology, China. Metabolites were dissolved in DMSO or water according to its solubility. For the compounds dissolved in water and the control group, an equal amount of DMSO was added. For metabolites, cells were plated on 6-well plates 24 hours prior to treatment and cultured with metabolites (1 mM) for 48 hours. Then, the supernatants from treated CRC cells were subjected to Enzyme-linked

immunosorbent assays.

For 4-HPA, concentration gradient experimentally, cells were plated on 6-well plates 24 hours prior to treatment and cultured with 4-HPA (0, 10 nM, 10 μM, 1 mM and 10 mM) for 48 hours; Time gradient experimentally, cells were plated on 6-well plates 24 hours prior to treatment and cultured with 4-HPA(1 mM) for (0, 24 h, 48 h, 72 h, 96 h). Then, the supernatants from treated CRC cells were subjected to Enzyme-linked immunosorbent assays.

RNA isolation, reverse transcription and real-time quantitative PCR (qPCR)

Total RNA was extracted using TRIzol Reagent according to the manufacturer's protocol and our previous report(1). The qPCR was carried out using TransStart Tip Green qPCR SuperMix (TransGen Biotech) on an ABI 7500HT system. GAPDH was chosen as an endogenous control. All primers were synthesized by Sangon (Supplementary Table 3). The expression level of each target gene was normalized as the fold change of the control gene or reference group expression. Fold changes were calculated through relative quantification ($2^{-\Delta\Delta}C^{t}$).

Enzyme-linked immunosorbent assays

The supernatants from treated CRC cells were collected and diluted for *Cxcl3* measurement using a mouse *Cxcl3* ELISA (enzyme-linked immunosorbent assay) Kit (Elabscience, #E-EL-M0147). Briefly, 100 μl of samples were added to appropriate wells, incubation at 37°C for 90 min, followed by the addition of 100 μl of antibody cocktail to each well, after incubation at 37 °C for 1 h. Each well was washed 3 times with 350 μl of wash buffer. 100 μl ABC solution was added and incubated for 30 min at 37 °C. Each well was washed 5 times with 350 μl of wash buffer. TMB solution was added and incubated for 15 min at 37 °C before the addition of

50 μl of stop solution prior to measurement of the absorbance at 450 nm.

Cell transfection

The full-length cDNAs of mouse *Cxcl3* were cloned and ligated into a pReceiver-M14 Expression clone vector. The short hairpin–mediated RNA (shRNA) sequences targeting mouse *Ccl20* and *Cxcl3* to knock down its expression (Supplementary Table 4). These shRNAs were cloned into a lentiviral transfer vector(psi-LVRU6P).

ChIP assays

ChIP was performed as we described previously(2). Eluted DNA was purified using Agencourt AMPure beads. For ChIP-PCR assays, the length of the product was identified by electrophoresis of the product after PCR with an agarose gel containing Gold View chromogenic agent.

Duel-luciferase reporter assay

The wild-type and mutated promoter segment sequences were synthesized and inserted into a pGL3-basic vector (Promega, USA). 293T cells were plated in 24-well plates at 5×10^3 cells per well 8 h before transfection. The cells were cotransfected with a mixture of 500 ng pGL3-basic-CXCL3 promoter, 100 ng Renilla and 500 ng pcDNA 3.0-STAT3 or control. Forty-eight hours later, the luciferase activity was measured using the Dual-Luciferase Reporter Assay System (Promega, USA).

EdU assays

CRC cells (1×10^6 cells per well) were plated onto 6-well plates. After 24 h, the cells were treated with 4-HPA (0, 10 nM, 10 μM, 1 mM and 10 mM). After incubation for 24 h, the cell

culture medium in each well was replaced with 1 mL of EdU medium (50 μM) and incubated for 2 h. The cells were harvested by trypsinization, and cell proliferation activities were determined using a Cell-Light™ EdU Apollo® 488 InVitro Flow Cytometry Kit (20T) (RiboBio, C10338-3).

HDAC Activity Detection

Histone deacetylase (HDAC) activity was measured by the HDAC Activity assay kit (Fluorometric; Abcam, ab156064) according to the manufacturer's instructions. according to the manufactory's protocol. 1×10^7 cells were washed and resuspended in lysis buffer (10 mM Tris HCl (pH 7.5),10 mM NaCl, 15 mM $MgCl_2$, 250 mM Sucrose. 0.5% NP-40, 0.1 mM EGTA) and lysed on ice for 15 min. Cells were centrifuged through 4 mL of sucrose cushion at 1300 ×g for 10 min at 4 °C. Pellet were washed and resuspended inextraction buffer (50 mM Hepes KOH (pH 7.5), 420 mM NaCl, 0.5 mM EDTA Na2, 0.1 mM EGTA, 10% glycerol) and then sonicated. Nuclei were lysed for 30 min and centrifuged at 20,000 ×g for 30 min. The enzymatic activity was assessed by measuring the fluorescence intensity of AMC at an excitation/emission wavelength of 355 nm/460 nm.

References

1. Wu L, et al. Calcium Channel Blocker Nifedipine Suppresses Colorectal Cancer Progression and Immune Escape by Preventing NFAT2 Nuclear Translocation (vol 33, 108327-1, 2020). *Cell Rep.* 2020;33(13).

2. Zhang F, et al. miR-589 promotes gastric cancer aggressiveness by a LIFR-PI3K/AKT-c-Jun regulatory feedback loop. *J Exp Clin Cancer Res.* 2018;37(1):152.

Supplementary Figures

S1A

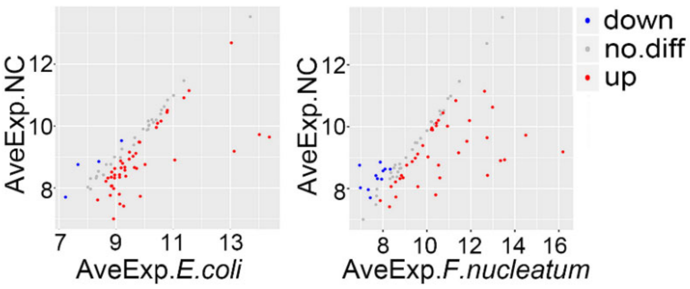

Supplementary Figure 1, related to Figure 1. Gut microbiota induced cytokine secretion in CRC cells. (A). Different cytokines and chemokines stimulated by *Fusobacterium* and *Escherichia-shigella* in CRC cells.

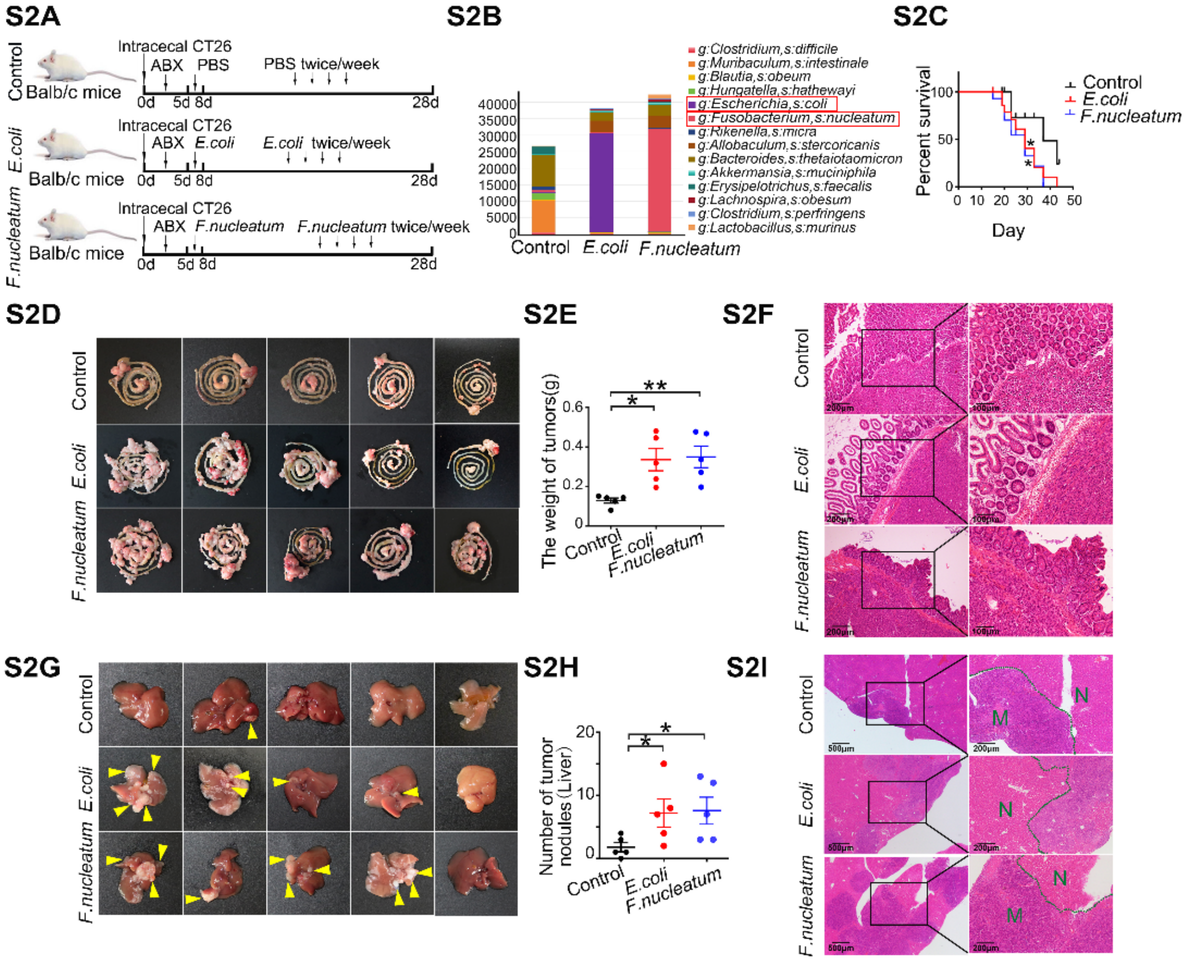

Supplementary Figure 2, related to Figure 2. Imbalance of intestinal microbiota mediates immunosuppressive microenvironment in CRC. (A-I). Results from the orthotopic implanted CRC mice model. **(A).** Schematic diagram of the orthotopic implanted CRC mice model administration method. **(B).** Metagenomic sequencing analysis of fecal samples from orthotopic implanted CRC mice. The relative abundance of OTUs is shown. **(C)** Survival analysis of orthotopic implanted CRC mice. **(D).** Tumors in situ of intestine in CT26 orthotopic CRC mice. Representative tumor images are shown (n=5). **(E).** Number of orthotopic tumors were measured(n=5). **(F).** HE staining of CRC in mice. **(G).** Liver metastases were determined. Representative images are shown (n=5). **(H).** Number of liver metastases nodules were

measured (n=5). **(I).** HE staining showed liver metastases. M stands for tumor metastasis; N stands for normal liver tissue. Data represent the mean ±SD of 3 independent experiments. Statistical analyses were conducted using 1-way ANOVA with Dunnett's correct multiple-comparison test. Survival curves were performed using the Mantel-Cox test. (*$p<0.05$).

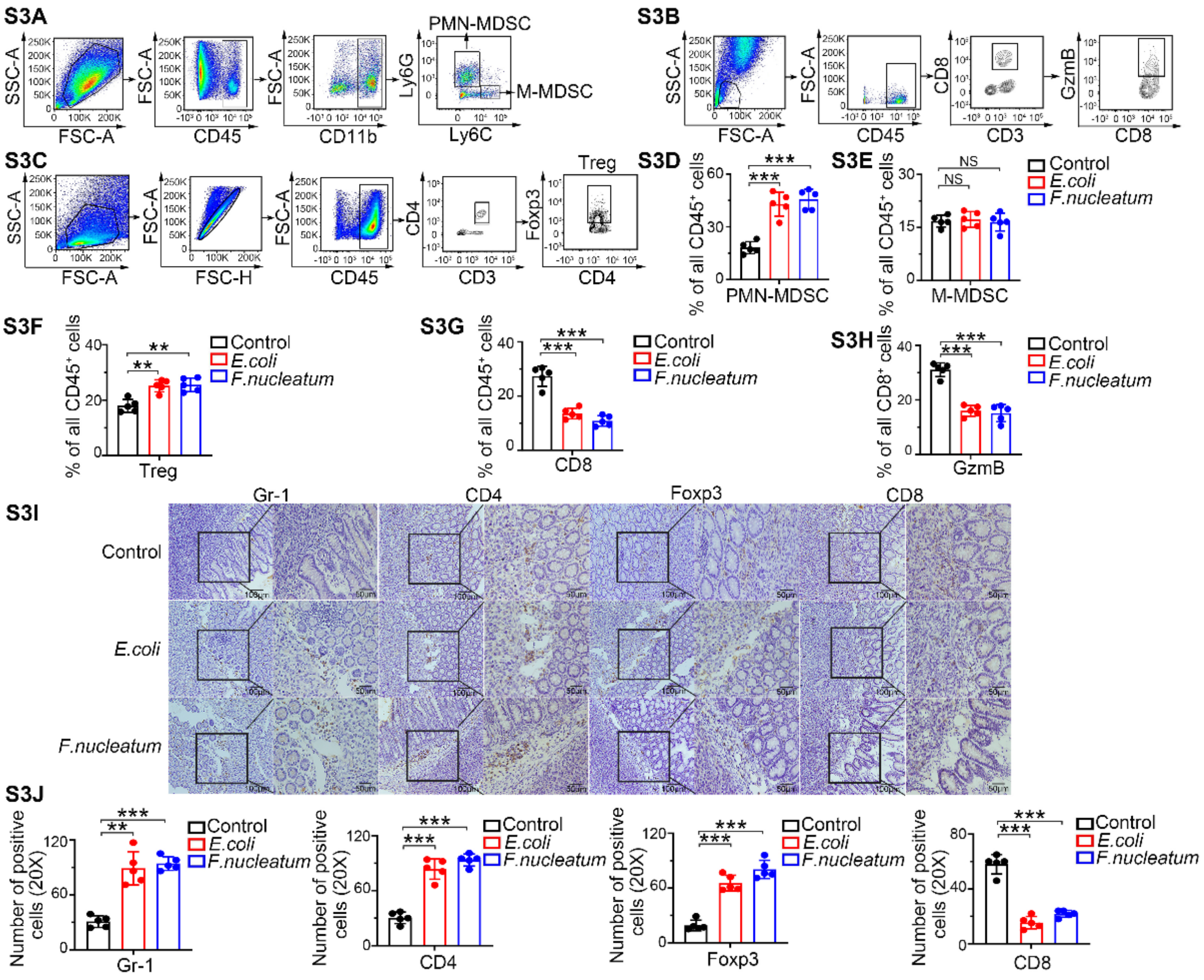

Supplementary Figure 3, related to Figure 2. Imbalance of intestinal microbiota mediates immunosuppressive microenvironment in CRC. (A). Flow Cytometry Gating Strategy for Tumor-Infiltrating Myeloid-Derived Suppressor cells (MDSCs). **(B).** Flow Cytometry Gating Strategy for Tumor-Infiltrating CD8^{+} T cells. **(C).** Flow Cytometry Gating Strategy for Tumor-Infiltrating Regulatory T Cells (Tregs). **(D-J).** Results from the orthotopic implanted CRC mice model**. (D)** Statistical chart of PMN-MDSCs (CD11b^{+}Ly6G^{+}Ly6C^{low}) and **(E)** M-MDSCs (CD11b^{+}Ly6G^{-}Ly6C^{hi}) in TILs (CD45^{+}) of mice detected by flow cytometry sorting (n=5). **(F).** Statistical chart of Tregs (CD4^{+}Foxp3^{+}) in TILs (CD45^{+}) of mice detected by flow cytometry sorting (n=5). **(G-H).** Statistical chart of Tumor-infiltrating CD8^{+}T cells and granule

productions ($GzmB^+$) in TILs ($CD45^+$) of mice detected by flow cytometry sorting (n=5). **(I).** MDSCs ($Gr\text{-}1^+$), Tregs ($CD4^+Foxp3^+$) and $CD8^+$ T cell infiltration in tumor tissues of mice. Representative IHC images are shown. **(J).** Histogram shows the number of $Gr\text{-}1^+$, $CD4^+$, $Foxp3^+$ and $CD8^+$ cells per 20× objective lens visual field (n=5). Data represent the mean ±SD of 3 independent experiments. Statistical analyses were conducted using 1-way ANOVA with Dunnett's T3 correct multiple-comparison test. (NS: not significantly different; *$p<0.05$; **$p<0.005$; ***$p<0.0005$).

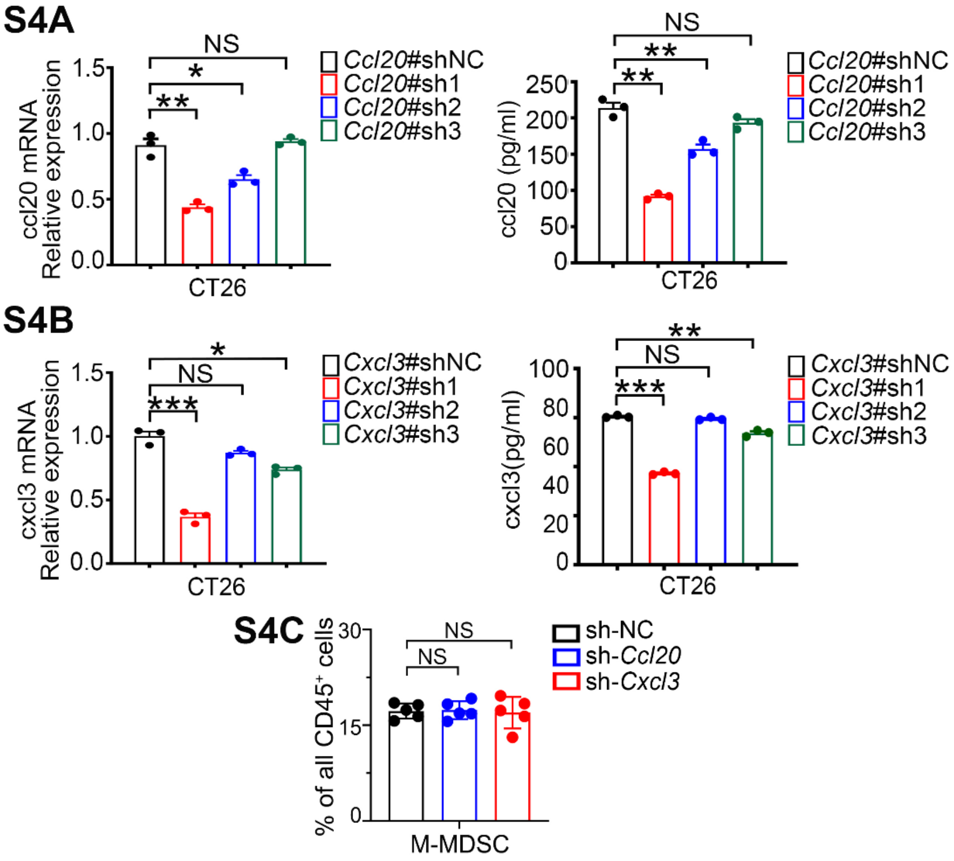

Supplementary Figure 4, related to Figure3. CXCL3 is the key cytokine of tumor immunosuppression caused by microbiota dysbiosis in CRC. (A). qRT-PCR and ELISA assays were performed to verify the successful construction of *Ccl20* knockdown CT26 cells (n=3). **(B).** qRT-PCR and ELISA assays were performed to verify the successful construction of *Cxcl3* knockdown CT26 cells (n=3). **(C).** Statistical chart of M-MDSCs in tumors of CT26 orthotopic implanted CRC mice detected by flow cytometry sorting (n=5). Data represent the mean ±SD of 3 independent experiments. Statistical analyses were conducted using 1-way ANOVA with Dunnett's T3 correct multiple-comparison test. (NS: not significantly different; *p<0.05; **p<0.005; ***p<0.0005).

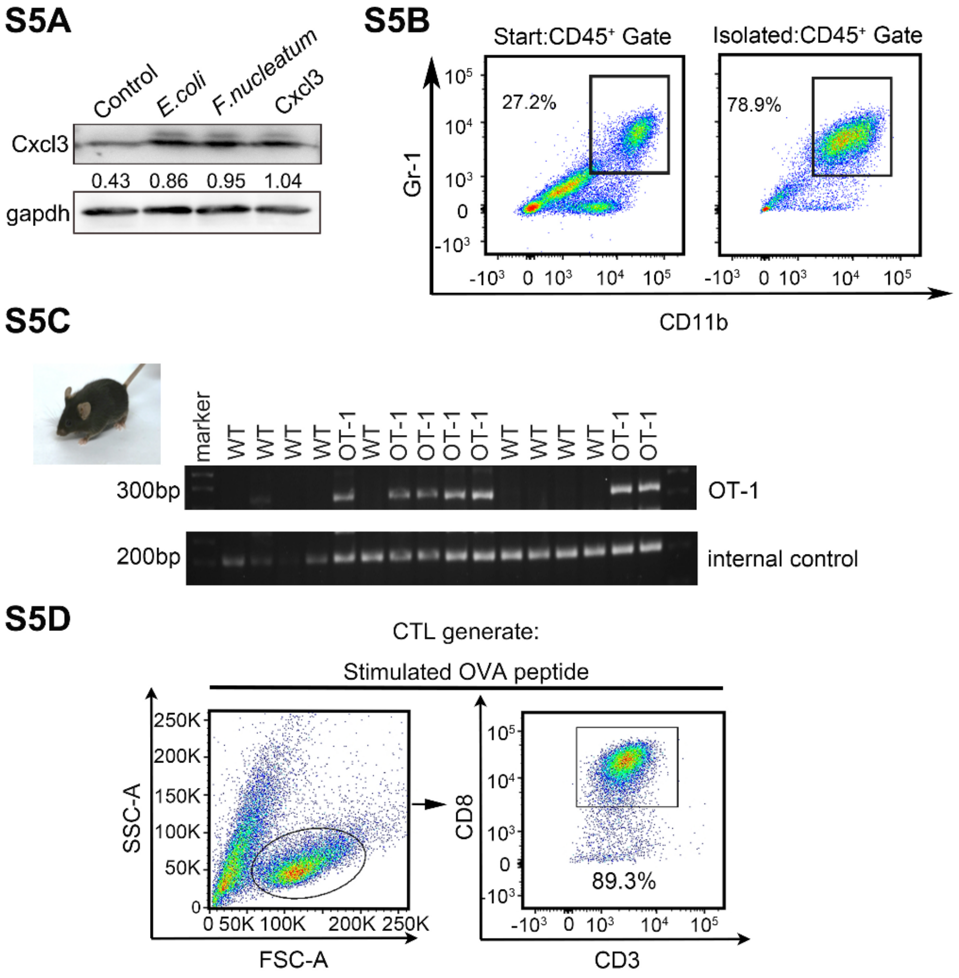

Supplementary Figure 5, related to Figure4. CXCL3/CXCR2 axis mediates MDSCs recruitment and inhibits T-cell effector function. (A). Western Blot analysis was used to detect *Cxcl3* in CT26 cells of the control group, *E.coli*, *F.nucleatum* and *Cxcl3*-overexpression group. **(B).** MDSCs isolation identification. **(C).** Genotype identification of OT-1 mice. **(D).** The generation of CTL. All the experiments were independently repeated at least 3 times.

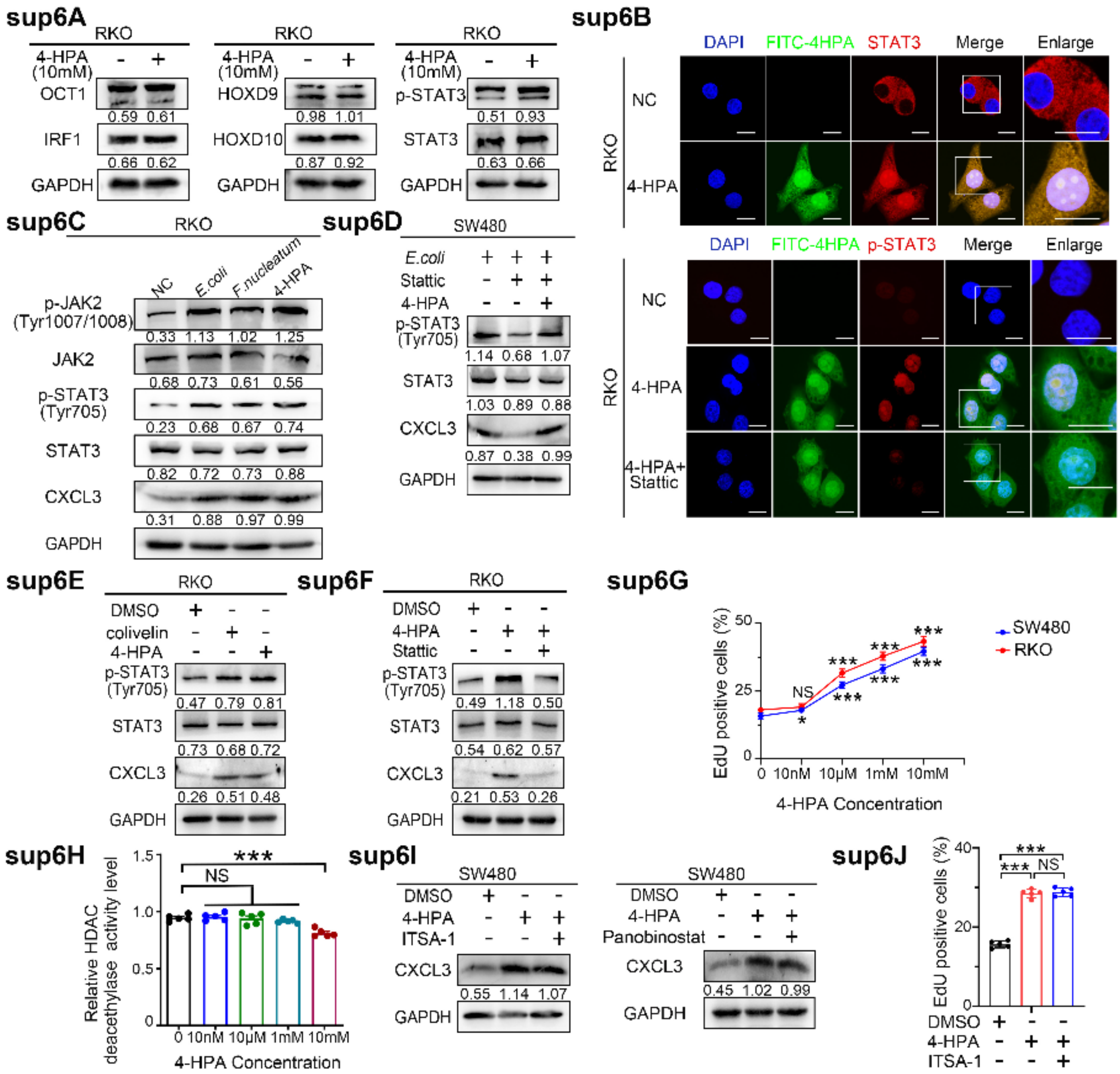

Supplementary Figure 6, related to Figure6. 4-HPA promotes the transcriptional regulation of CXCL3 by STAT3. (A). Western Blot analysis of the effects of 4-HPA on transcription factors in RKO cells. **(B).** Immunofluorescence (IF) assays were used to visualize the subcellular localization of STAT3 and phosphorylated; STAT3 in RKO cells treated with 4-HPA or Stattic. Scale bar: 50 μm. **(C).** Western Blot analysis was used to detect the effects of 4-HPA on the JAK2/STAT3 signaling pathway in CRC cells. **(D-F).** Western Blot analysis was used to detect the effects of 4-HPA on CXCL3 in CRC cells. **(G).** CRC cells

were treated with 4-HPA (0, 10 nM, 10 μM, 1 mM and 10 mM) for 24 h, and proliferation activities were evaluated via EdU (n=5). **(H).** HDACs activity of SW480 cells treated with 4-HPA (0, 10 nM, 10 μM, 1 mM and 10 mM) for 24 h (n=5). **(I).** Western Blot analysis was used to detect the CXCL3 in DMSO group, 4-HPA (1 mM) with or without HDAC activator ITSA-1(50 μM) group and 4-HPA(1 mM) with or without HDAC inhibitor Panobinostat (50 nM) group in SW480 cells. **(J).** EdU analysis was used to detect the proliferation ability in SW480 cells in DMSO group, 4-HPA(1 mM) with or without HDAC activator ITSA-1(50 μM) group. Data represent the mean ±SD of 3 independent experiments. Statistical analyses were conducted using 1-way ANOVA with Dunnett's T3 correct multiple-comparison test. (NS: not significantly different; *p<0.05; **p<0.005; ***p<0.0005).

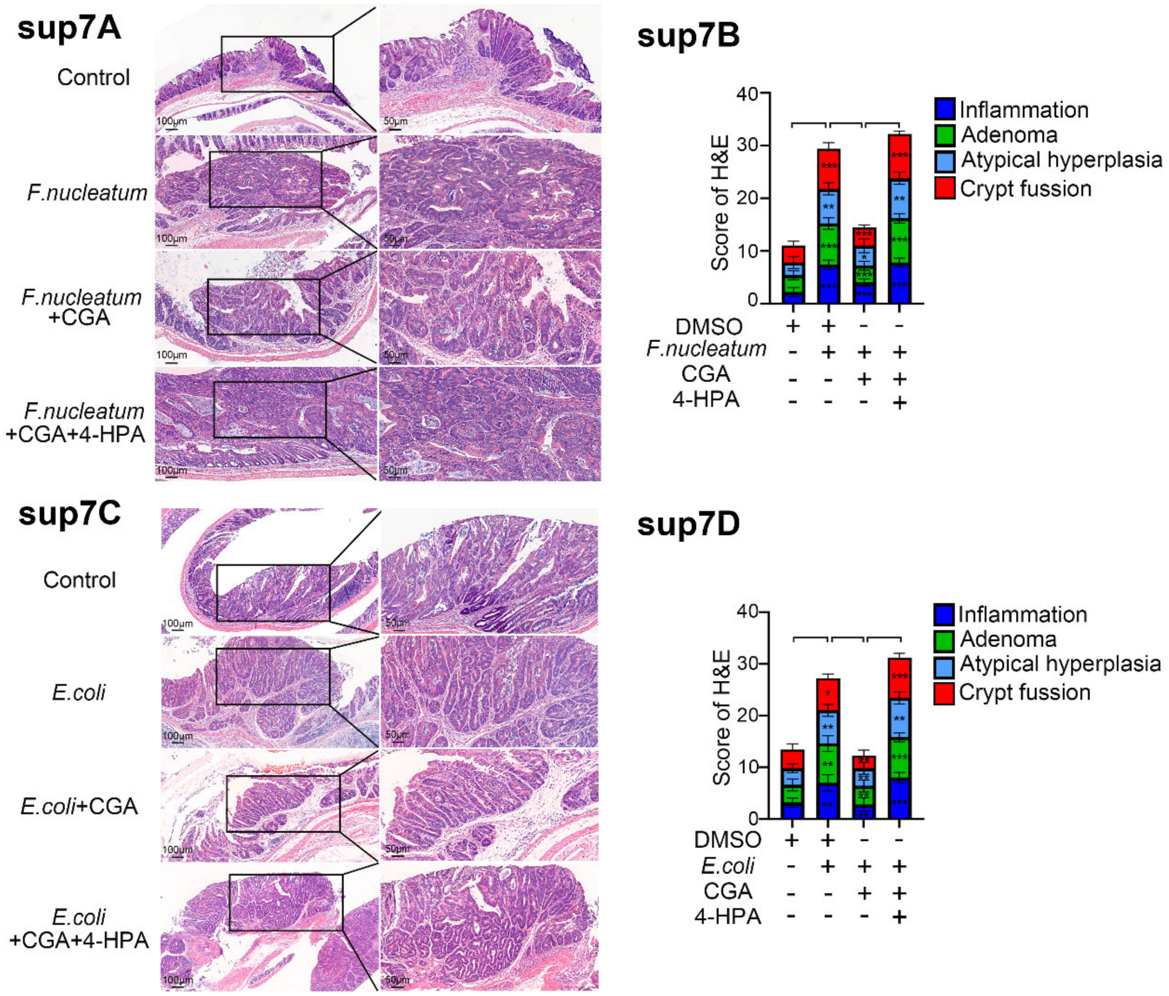

Supplementary Figure 7, related to Figure7. 4-HPA correlates with PMN-MDSC accumulation in CRC. (A). HE of intestines derived from Apc$^{min/+}$ mice colonized with *F.nucleatum* and fed with or without 4-HPA (1 mM) and CGA (1 mM). Representative images were displayed. **(B).** HE scoring of tumor-related lesions (including inflammation, adenoma, atypical hyperplasia and crypt fusion) (n=5). **(C).** HE of intestines derived from Apc$^{min/+}$ mice colonized with *E.coli* and fed with or without 4-HPA (1 mM) and CGA (1 mM). Representative images were displayed. **(D).** HE scoring of tumor-related lesions (including inflammation, adenoma, atypical hyperplasia and crypt fusion) (n=5). Data represent the mean ±SD of 3

independent experiments. Each lesion statistical analyses were conducted using 1-way ANOVA with Dunnett's T3 correct multiple-comparison test. (**$p<0.005$).

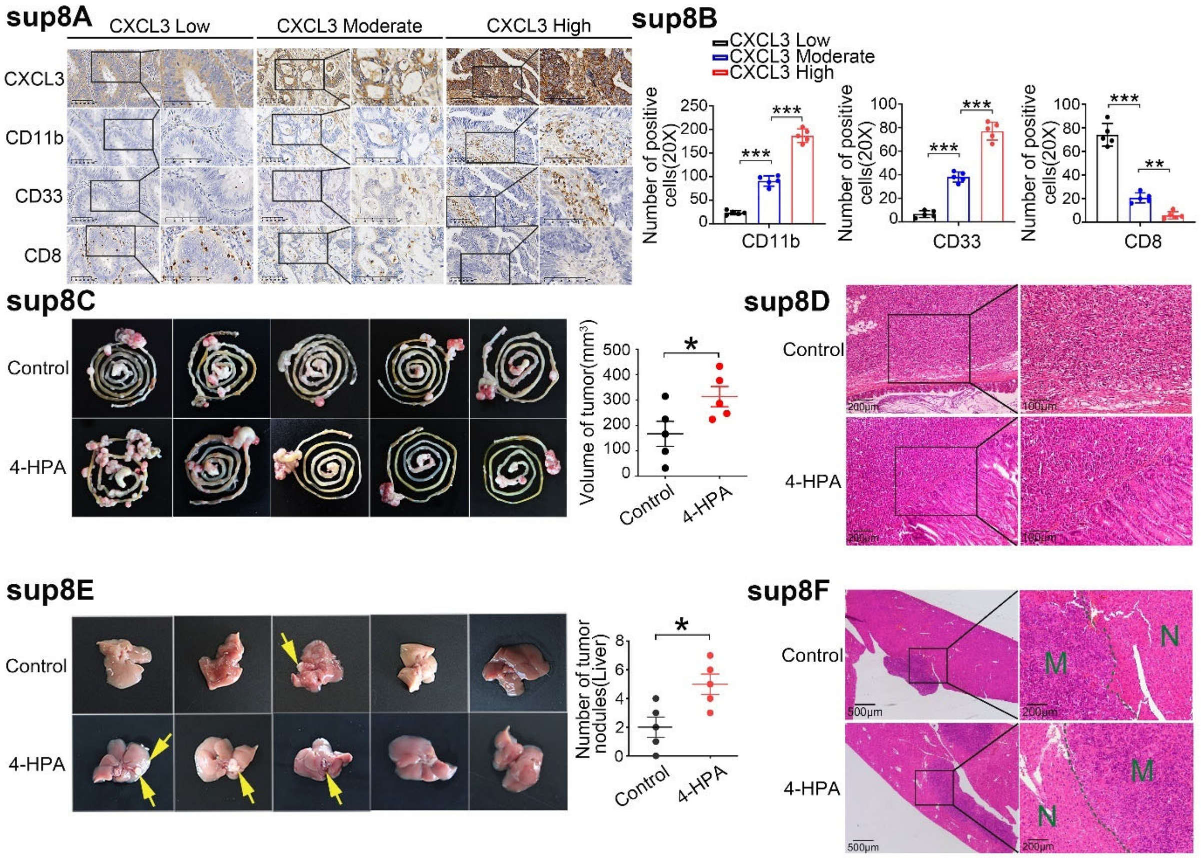

Supplementary Figure 8, related to Figure7. 4-HPA correlates with PMN-MDSC accumulation in CRC. (A). Immunohistochemical detection of the correlation between CXCL3, CD8, $CD33^+$ and $CD11b^+$ cells in tumor tissue. Visualization of three representative cases is shown. **(B).** The histogram shows the number of $CD11b^+$, $CD33^+$ and $CD8^+$T cells per 20× objective lens visual field (n=5). **(C).** Tumors in situ of intestine in CT26 orthotopic implanted CRC mice fed with 4-HPA (1 mM) or control water (DMSO). Representative tumor images are shown. Volume of tumors in situ of intestine was measured (right) (n=5). **(D).** HE staining revealed CRC in mice. **(E).** Liver metastases were observed. Representative images are shown. Number of liver metastatic nodules was measured (right) (n=5). **(F).** HE staining revealed liver metastasis. M stands for tumor metastasis; N stands for normal liver tissue. Data

represent the mean ±SD of 3 independent experiments. Statistical analyses were conducted using student's t-test (two-comparison test) and 1-way ANOVA with Dunnett's T3 correct multiple-comparison test. (*, $p<0.05$; **, $p<0.005$; ***, $p<0.0005$).

Supplementary Table 1. Panel of inflammatory cytokine arrays (AAH-CYT-G5-4). Detect 80 Cytokines, Chemokines and Growth Factors in One Experiment.

	A	B	C	D	E	F	G	H	I	J	K	L	M
1	Pos 1	Pos 2	Pos 3	Neg	Neg	Neg	ENA-78	GCSF	GM-CSF	GRO	GRO-a	I-309	IL-1a
2	Pos 1	Pos 2	Pos 3	Neg	Neg	Neg	ENA-78	GCSF	GM-CSF	GRO	GRO-a	I-309	IL-1a
3	IL-1 b	IL-2	IL-3	IL-4	IL-5	IL-6	IL-7	IL-8	IL-10	IL-12 p70	IL-13	IL-15	IFN-g
4	IL-1 b	IL-2	IL-3	IL-4	IL-5	IL-6	IL-7	IL-8	IL-10	IL-12 p70	IL-13	IL-15	IFN-g
5	MCP-1	MCP-2	MCP-3	MCSF	MDC	MIG	MIP-1b	MIP-1d	RANTES	SCF	SDF-1	TARC	TGF-b1
6	MCP-1	MCP-2	MCP-3	MCSF	MDC	MIG	MIP-1b	MIP-1d	RANTES	SCF	SDF-1	TARC	TGF-b1
7	TNF-a	TNF-b	EGF	IGF-I	Angiogenin	Oncostatin M	Thrombopoietin	VEGF	PDGF-BB	Leptin	BDNF	BLC	Ck b 8-1
8	TNF-a	TNF-b	EGF	IGF-I	Angiogenin	Oncostatin M	Thrombopoietin	VEGF	PDGF-BB	Leptin	BDNF	BLC	Ck b 8-1
9	Eotaxin	Eotaxin-2	Eotaxin-3	FGF-4	FGF-6	FGF-7	FGF-9	Flt-3 Ligand	Fractalkine	GCP-2	GDNF	HGF	IGFBP-1
10	Eotaxin	Eotaxin-2	Eotaxin-3	FGF-4	FGF-6	FGF-7	FGF-9	Flt-3 Ligand	Fractalkine	GCP-2	GDNF	HGF	IGFBP-1
11	IGFBP-2	IGFBP-3	IGFBP-4	IL-16	IP-10	LIF	LIGHT	MCP-4	MIF	MIP-3a	NAP-2	NT-3	NT-4
12	IGFBP-2	IGFBP-3	IGFBP-4	IL-16	IP-10	LIF	LIGHT	MCP-4	MIF	MIP-3a	NAP-2	NT-3	NT-4

13	Osteopontin	Osteoprotegerin	PARC	PIGF	TGF- b 2	TGF- b 3	TIMP-1	TIMP-2	Neg	Neg	Neg	Neg	Neg
14	Osteopontin	Osteoprotegerin	PARC	PIGF	TGF- b 2	TGF- b 3	TIMP-1	TIMP-2	Neg	Neg	Neg	Neg	Neg

Supplementary Table 2. Participant characteristics of feces sampling.

	Discovery cohort		
	**CRC (n=20)**	**HC (n=20)**	**P values**
**Gender**			
**Female**	8 (40%)	11 (55%)	0.527
**Male**	12 (60%)	9 (45%)	
**Age**			0.25
**median years (min-max)**	53 (21-70)	49 (23-71)	
**BMI**			
**median kg/m^2(min-max)**	24.84	23.87	0.13
	15.67-31.78	17.45-29.43	
**Ever smoker**			
**No**	17 (85%)	18 (90%)	>0.9
**Yes**	3 (15%)	2 (10%)	
**Ever drinking**			
**No**	16 (80%)	15 (75%)	>0.9
**Yes**	4 (20%)	5 (25%)	

Abbreviations: BMI, body mass index; CRC: Colorectal cancer; HC, healthy control. Continuous variables were compared using Wilcoxon Rank Sum Test between groups. Fisher's exact test compared categorical variables.

Supplementary Table 3. Primers for RT-PCT analysis

Name	Primer (5'-3')	Primer (5'-3')
MIP3α	TGCTGTACCAAGAGTTTGCTC	CGCACACAGACAACTTTTTCTTT
MCP-1	CAGCCAGATGCAATCAATGCC	TGGAATCCTGAACCCACTTCT
GRO α	AAGAACATCCAAAGTGTGAACG	CACTGTTCAGCATCTTTTCGAT
GRO β	TGCTGCTCCTGCTCCTGGTG	GGGGACTTCACCTTCACACTTTGG
GROγ	CGCCCAAACCGAAGTCATAG	GCTCCCCTTGTTCAGTATCTTTT
MIF	TCGTAAACACCAACGTGCC	AGTTGATGTAGACCCTGTCCG
GM-CSF	TCCTGAACCTGAGTAGAGACAC	TGCTGCTTGTAGTGGCTGG
GAPDH	GGAGCGAGATCCCTCCAAAAT	GGCTGTTGTCATACTTCTCATGG

Supplementary Table 4. Sequences of shRNAs

Name	Location	Length (bp)	Target Sequence
Mus musculus Ccl20 -shRNA-1	219	21	CCGATGAAGCTTGTGACATTA
Mus musculus Ccl20 -shRNA-2	328	21	CCTAAGAGTCAAGAAGATGTA
Mus musculus Ccl20 -shRNA-3	646	21	GGGAATAGTTTATATGGACCT
Mus musculus Cxcl3-shRNA-1	187	21	GAACACCCTACCAAGGGTTGA
Mus musculus Cxcl3-shRNA-2	275	21	GCCACTCTCAAGGATGGTCAA
Mus musculus Cxcl3-shRNA-3	322	21	CAGGCTTCAGATAATCATCAA
