# Supplementary material for: Gut microbial metabolite 4-hydroxybenzeneacetic acid drives colorectal cancer progression via accumulation of immunosuppressive PMN-MDSCs
Source: J Clin Invest. 2025 Apr 3;135(11):e181243. doi: 10.1172/JCI181243 (PMC12126219; doi:10.1172/JCI181243)

Full unedited gel for Figure 6D

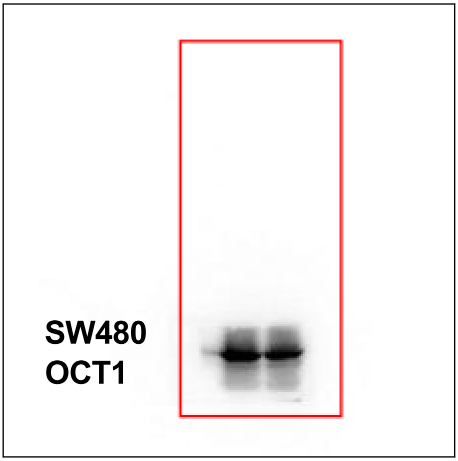

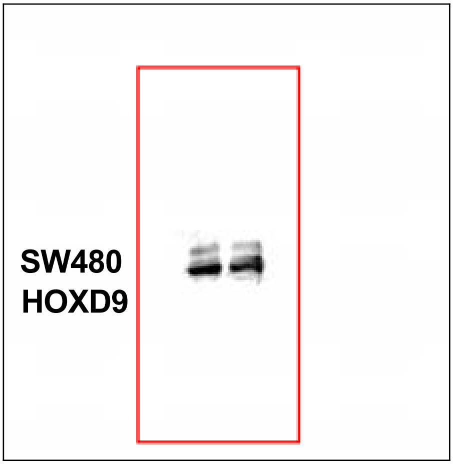

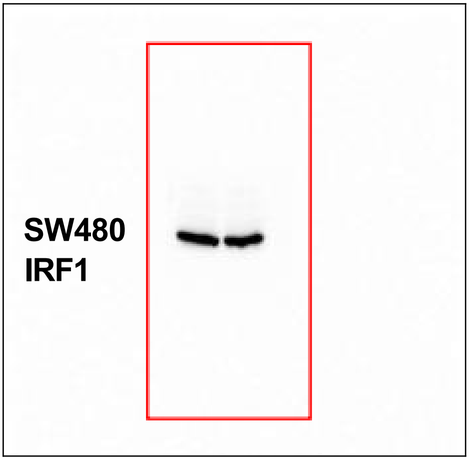

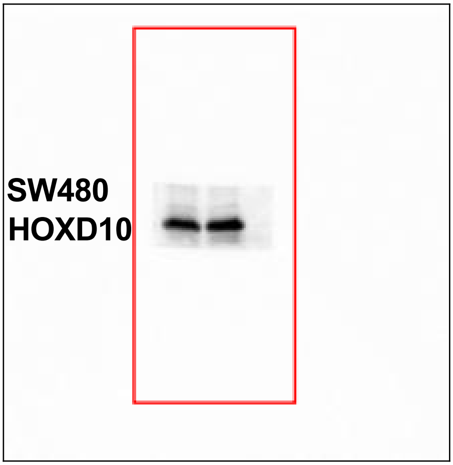

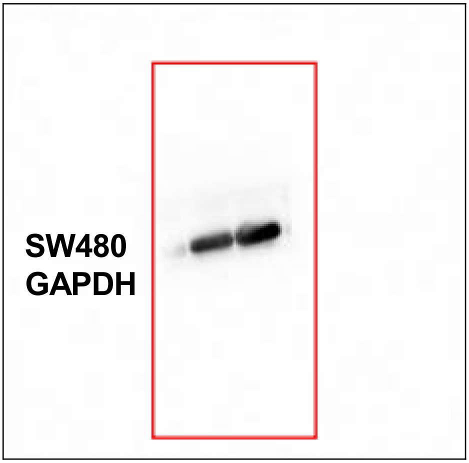

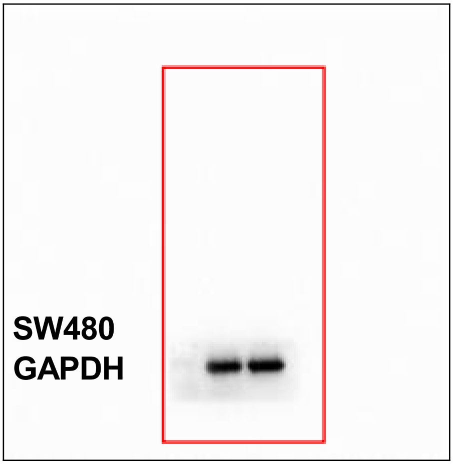

Full unedited gel for Figure 6D

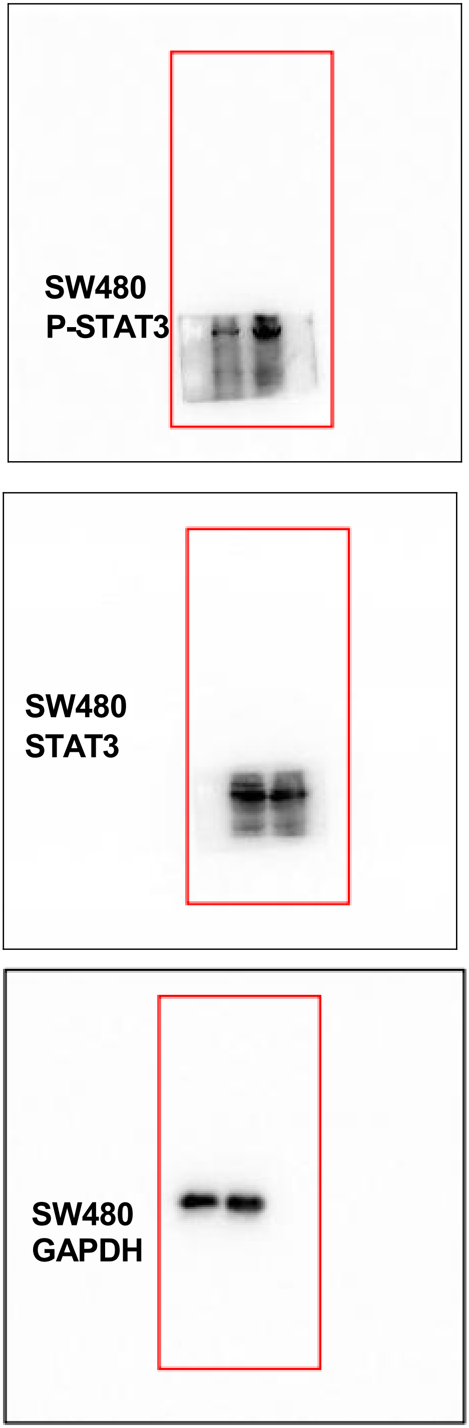

Full unedited gel for Figure 6G

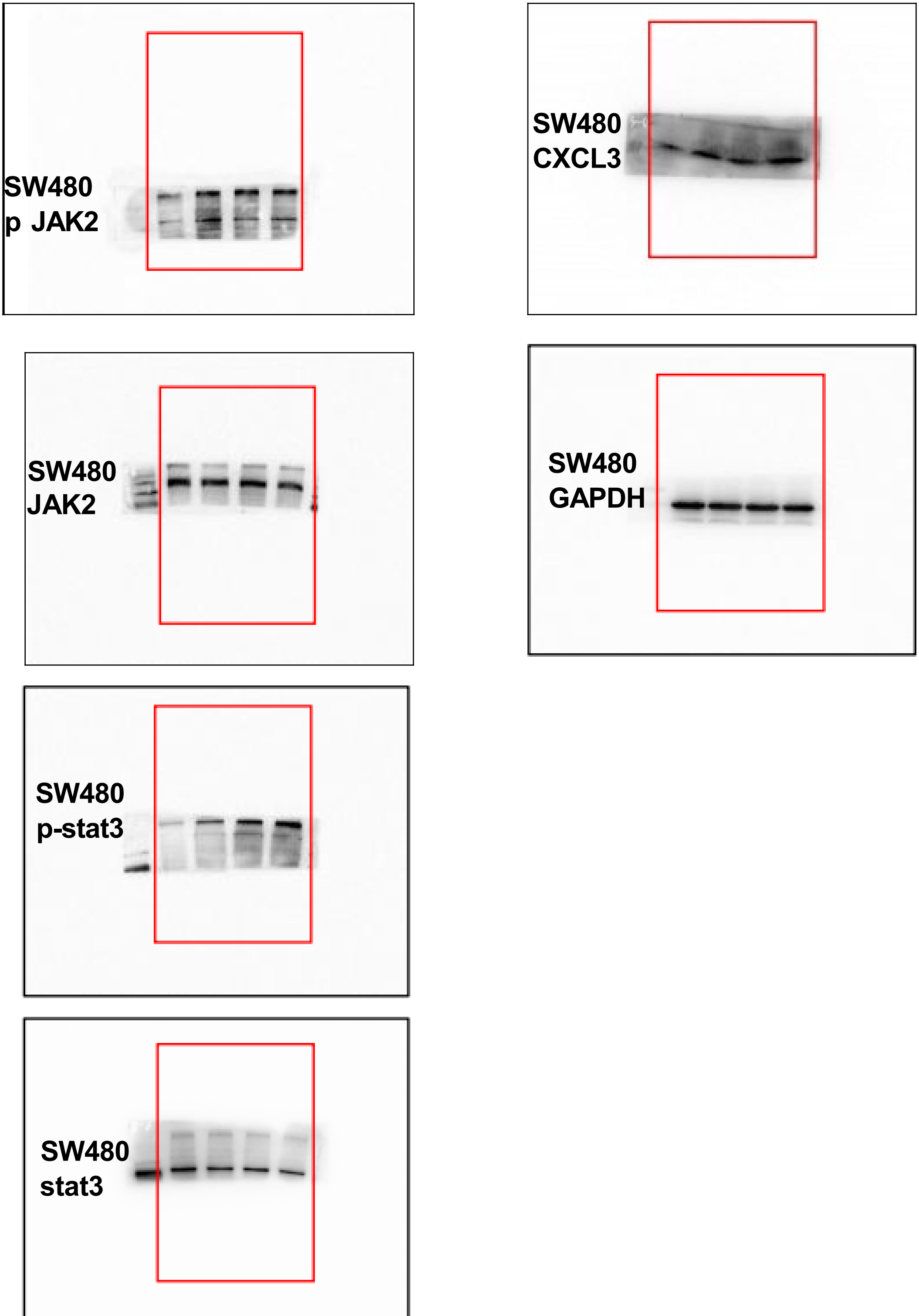

Full unedited gel for Figure 6H

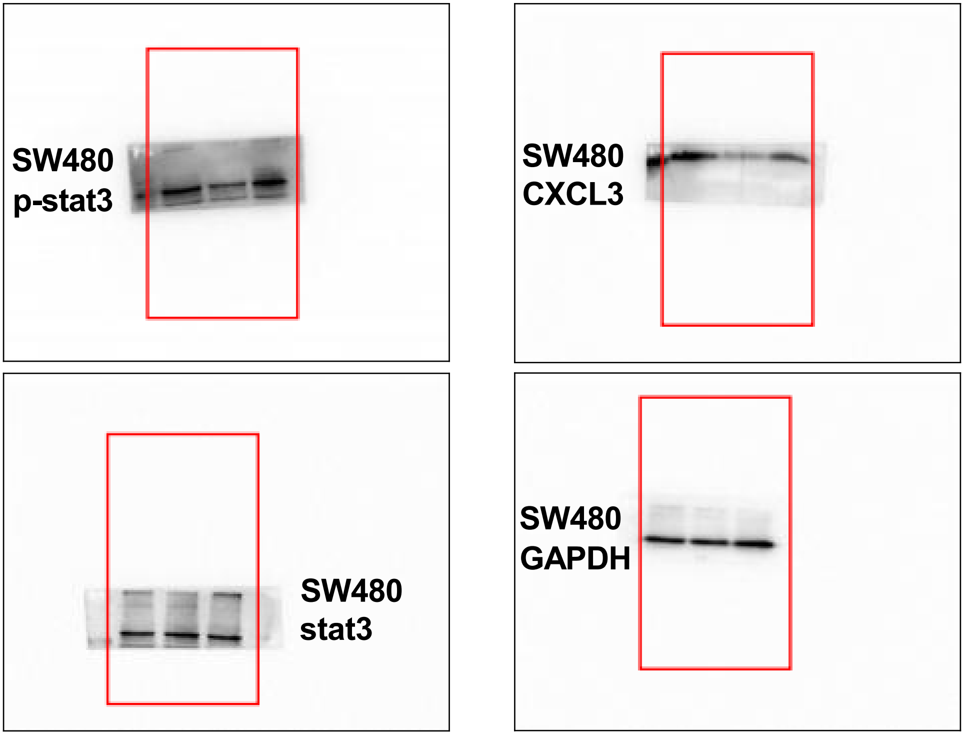

Full unedited gel for Figure 6I

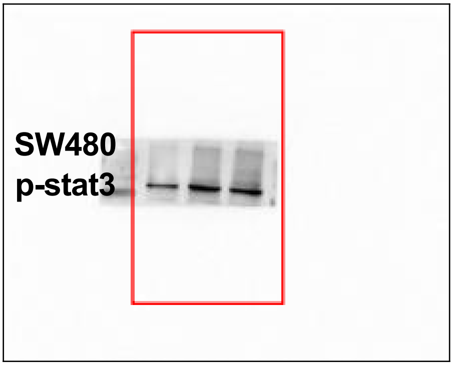

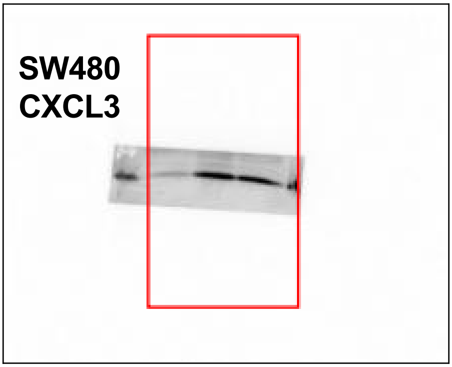

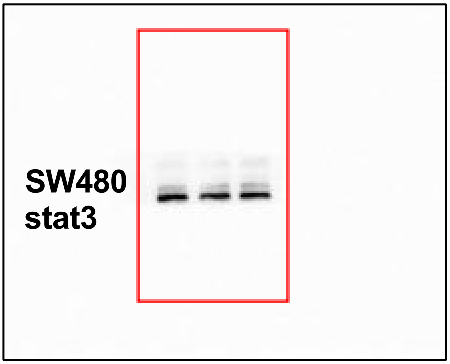

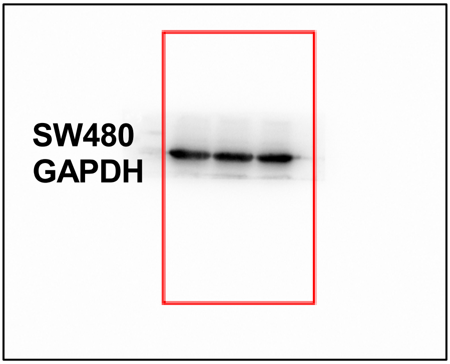

Full unedited gel for Figure 6J

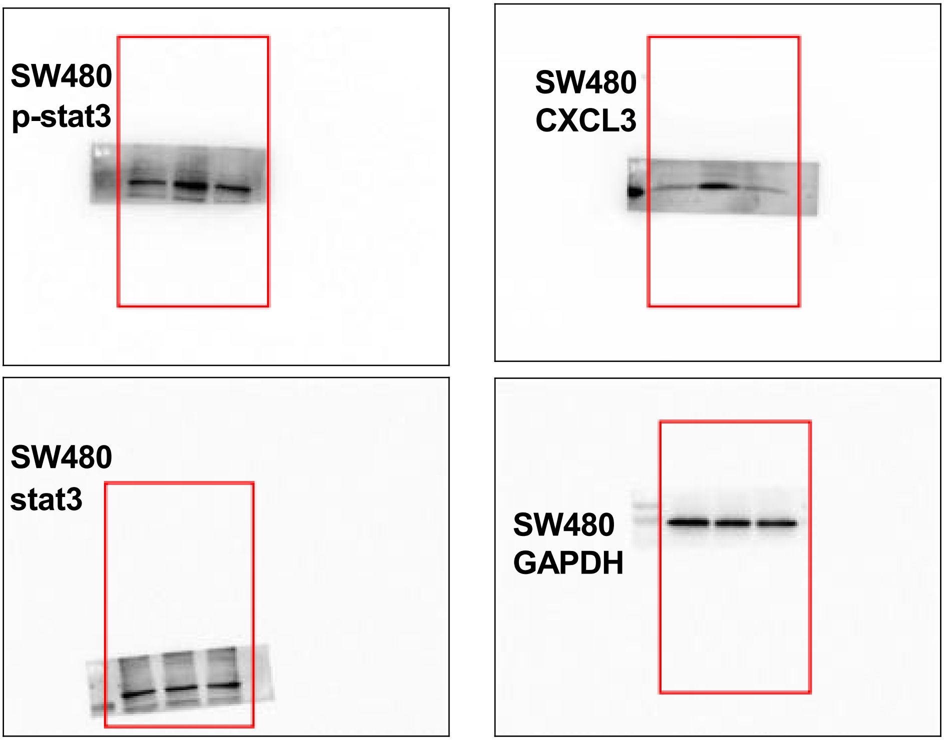

Full unedited gel for Figure S5A

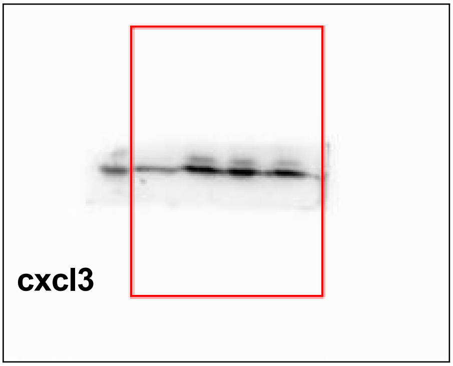

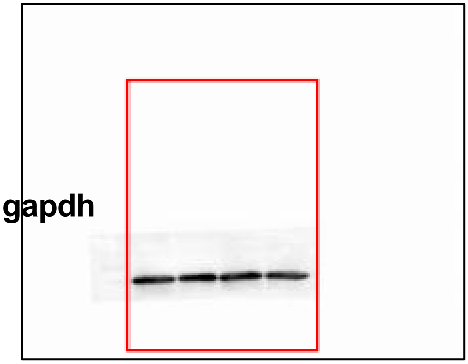

Full unedited gel for Figure S6A

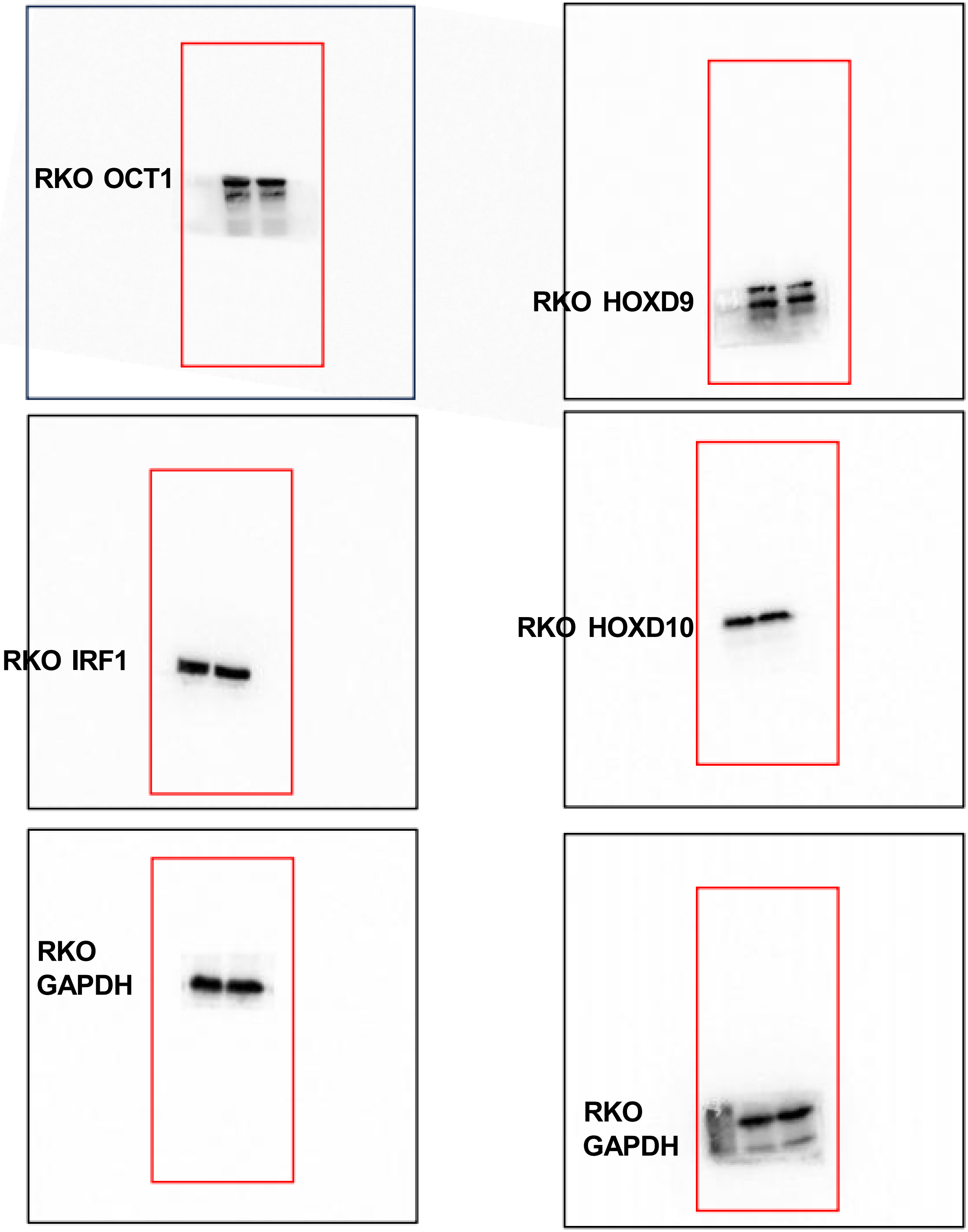

Full unedited gel for Figure S6A

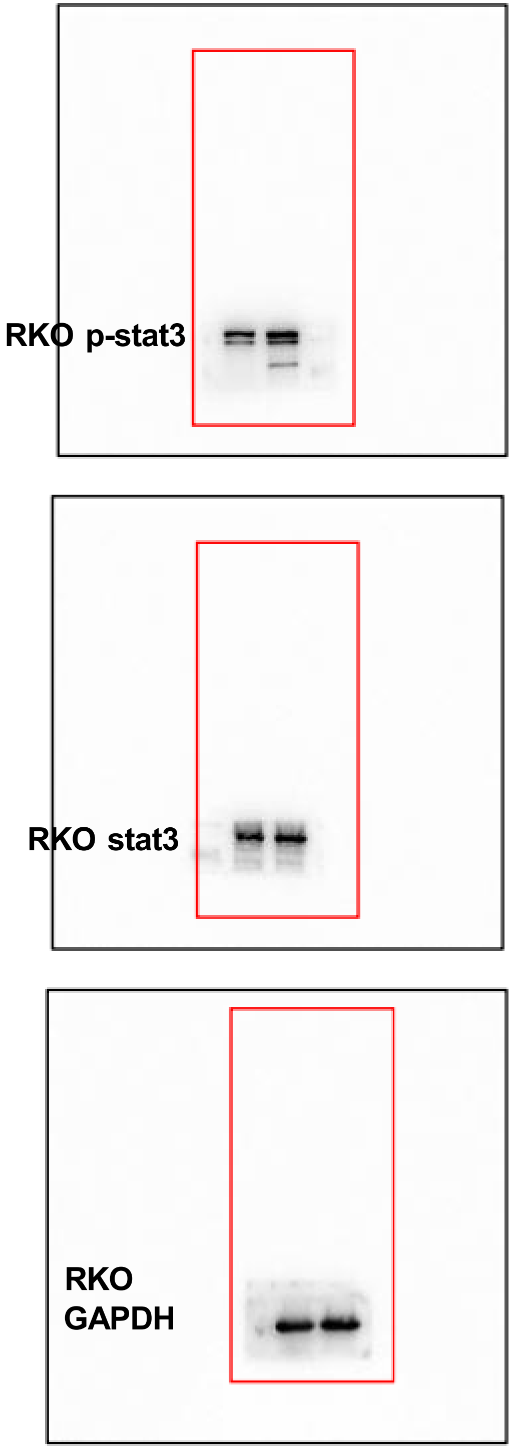

Full unedited gel for Figure S6C

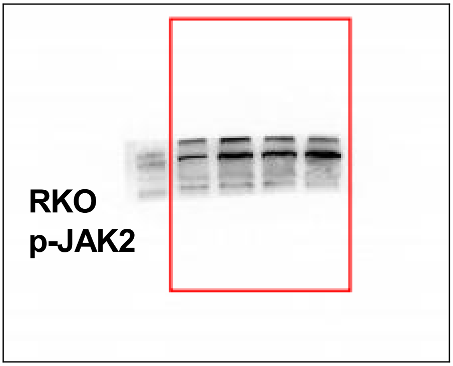

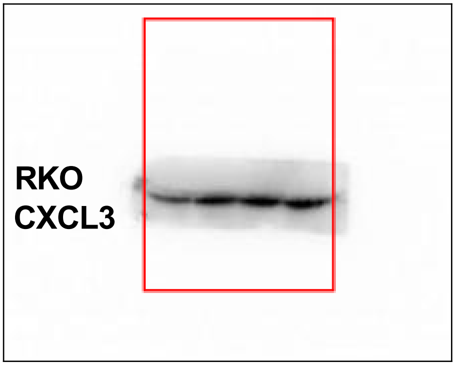

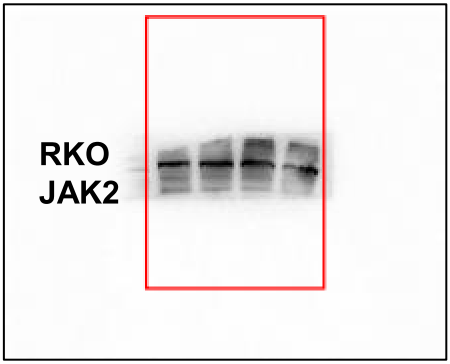

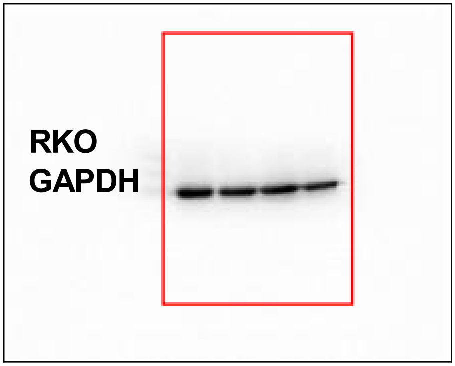

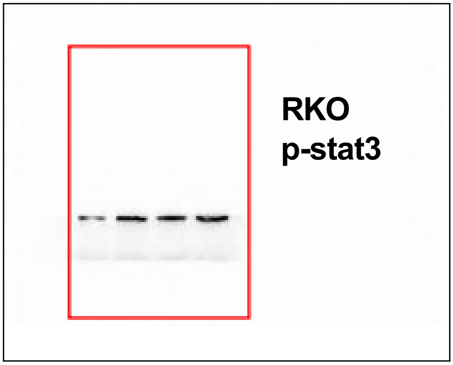

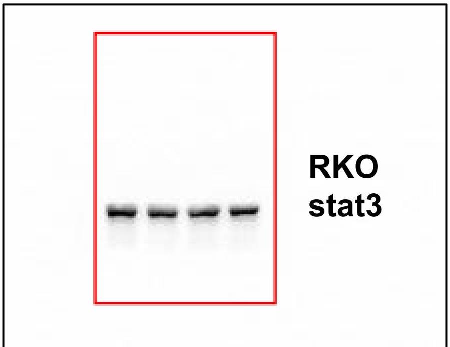

Full unedited gel for Figure S6D

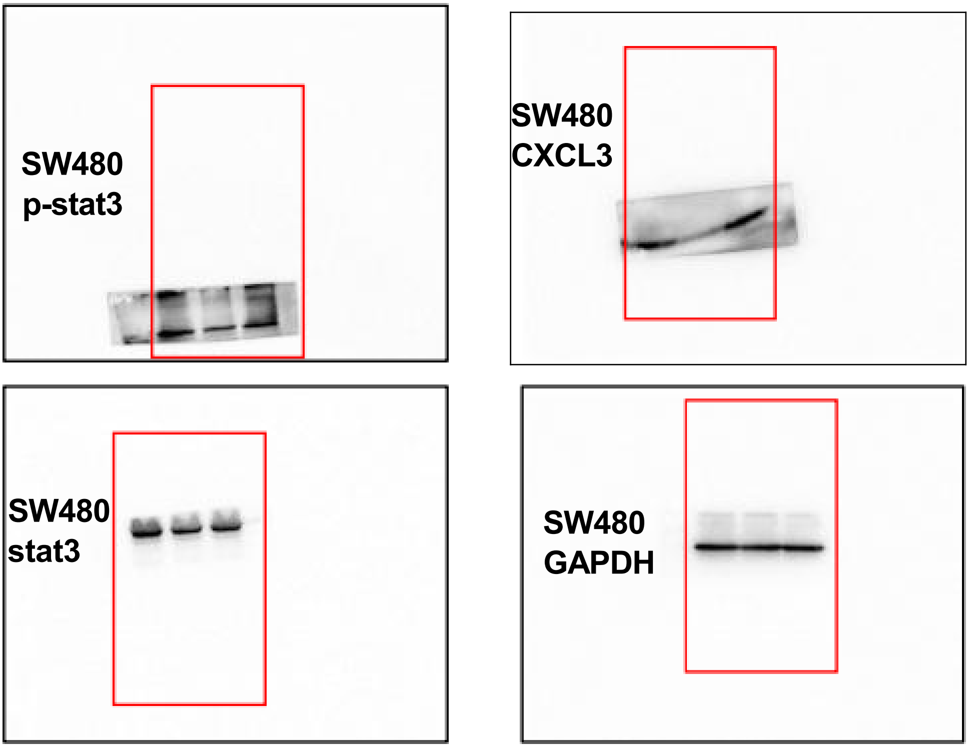

Full unedited gel for Figure S6E

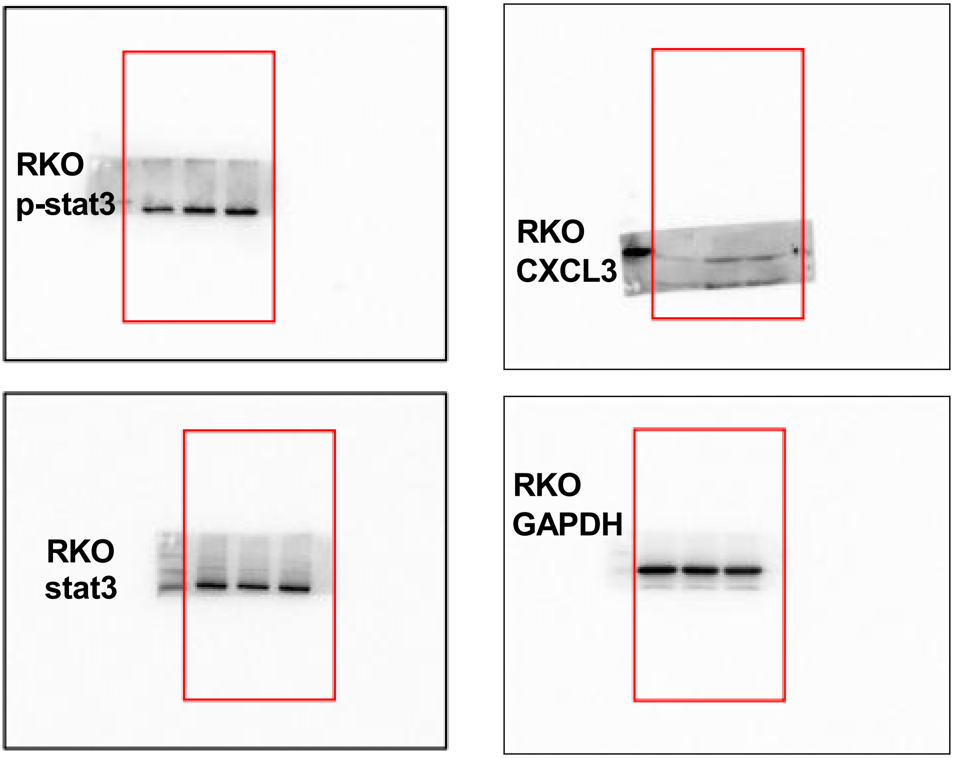

Full unedited gel for Figure S6F

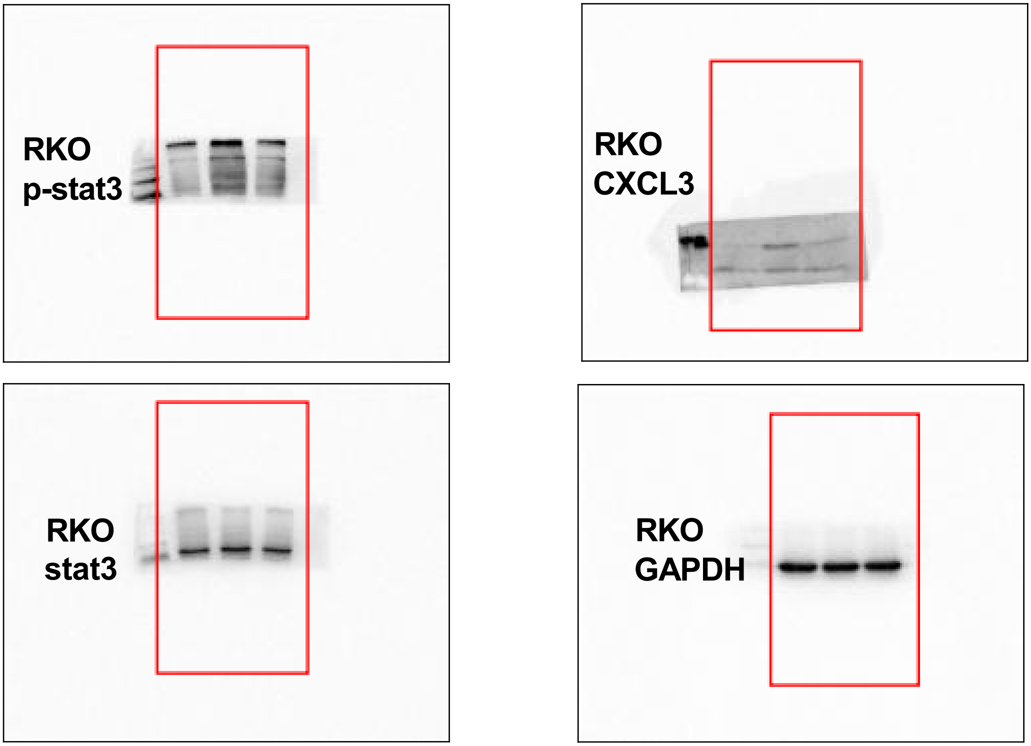

Full unedited gel for Figure S6I

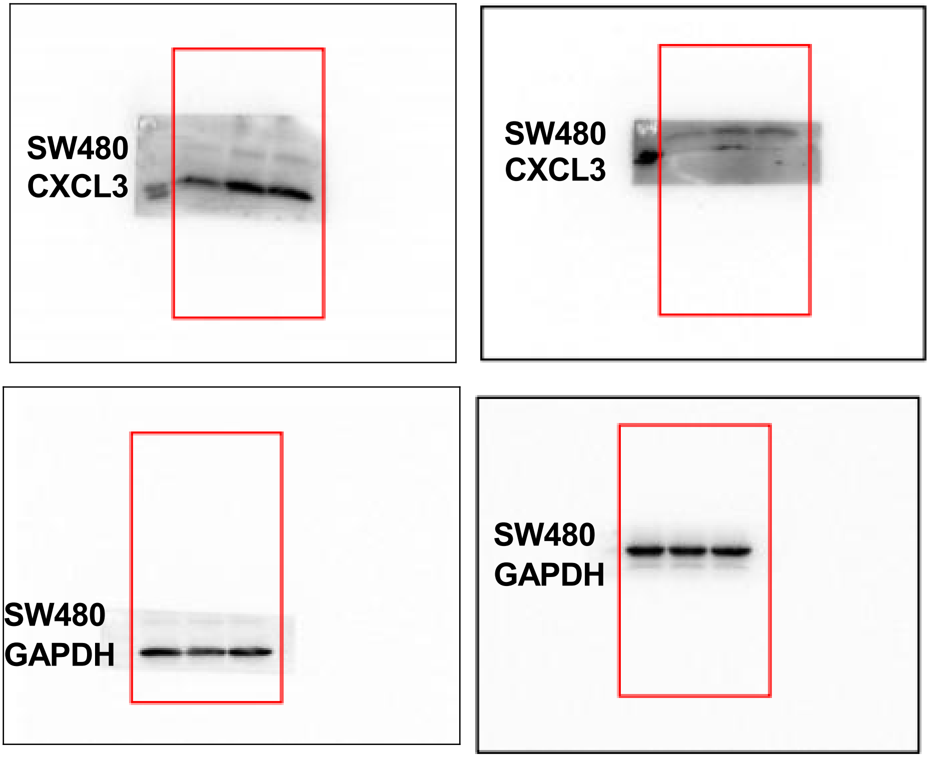

Supplement: Unedited blot and gel images [file jci-135-181243-s041.pdf]
